# Exploring Structural Uncertainty in Cost-Effectiveness Modeling of Gestational Diabetes Screening: An Application Example from Norway

**DOI:** 10.1177/0272989X241241339

**Published:** 2024-04-09

**Authors:** Pia S. Henkel, Emily A. Burger, Line Sletner, Kine Pedersen

**Affiliations:** Department of Health Management and Health Economics, University of Oslo, Oslo, Norway; Department of Health Management and Health Economics, University of Oslo, Oslo, Norway; Center for Health Decision Science, Harvard T.H. Chan School of Public Health, Boston, MA, USA; Department of Pediatric and Adolescents Medicine, Akershus University Hospital, Lørenskog, Norway; Department of Health Management and Health Economics, University of Oslo, Oslo, Norway

**Keywords:** cost-effectiveness analysis, cost-utility analysis, economic evaluation, screening, gestational diabetes mellitus

## Abstract

**Background:**

Screening pregnant women for gestational diabetes mellitus (GDM) has recently been expanded in Norway, although screening eligibility criteria continue to be debated. We aimed to compare the cost-effectiveness of alternative GDM screening strategies and explored structural uncertainty and the value of future research in determining the most cost-effective eligibility criteria for GDM screening in Norway.

**Design:**

We developed a probabilistic decision tree to estimate the total costs and health benefits (i.e., quality-adjusted life-years; QALYs) associated with 4 GDM screening strategies (universal, current guidelines, high-risk, and no screening). We identified the most cost-effective strategy as the strategy with the highest incremental cost-effectiveness ratio below a Norwegian benchmark for cost-effectiveness ($28,400/QALY). We excluded inconclusive evidence on the effects of screening on later maternal type 2 diabetes mellitus (T2DM) in the primary analysis but included this outcome in a secondary analysis using 2 different sources of evidence (i.e., Cochrane or US Preventive Services Task Force). To quantify decision uncertainty, we conducted scenario analysis and value-of-information analyses.

**Results:**

Current screening recommendations were considered inefficient in all analyses, while universal screening was most cost-effective in our primary analysis ($26,014/QALY gained) and remained most cost-effective when we assumed a preventive effect of GDM treatment on T2DM. When we assumed no preventive effect, high-risk screening was preferred ($19,115/QALY gained). When we assumed GDM screening does not prevent perinatal death in scenario analysis, all strategies except no screening exceeded the cost-effectiveness benchmark. In most analyses, decision uncertainty was high.

**Conclusions:**

The most cost-effective screening strategy, ranging from no screening to universal screening, depended on the source and inclusion of GDM treatment effects on perinatal death and T2DM. Further research on these long-term outcomes could reduce decision uncertainty.

**Highlights:**

- This article analyses the cost-effectiveness of 4 alternative gestational diabetes mellitus (GDM) screening strategies in Norway: universal screening, current (broad) screening, high-risk screening, and no screening.
- The current Norwegian screening recommendations were considered inefficient under all analyses.
- The most cost-effective screening strategy ranged from no screening to universal screening depending on the source and inclusion of GDM treatment effects on later maternal diabetes and perinatal death.
- The parameters related to later maternal diabetes and perinatal death accounted for most of the decision uncertainty.

## Graphical abstract

**Graphical Abstract:**
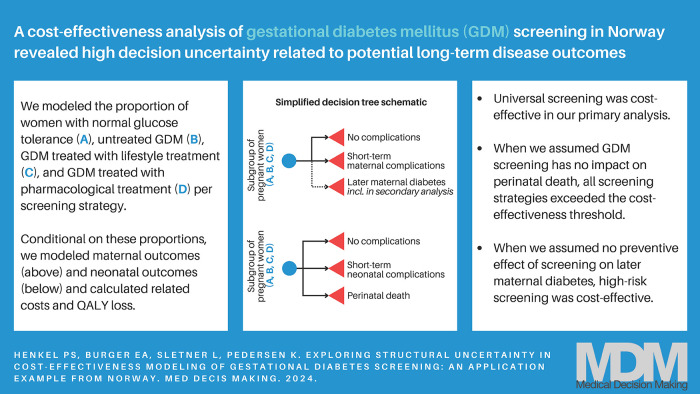
This is a visual representation of the abstract.

Gestational diabetes mellitus (GDM) describes impaired glucose tolerance during pregnancy and is considered the most frequent pregnancy complication.^1^ The degree of the resulting hyperglycemia (i.e., elevated blood glucose levels) can vary, but GDM is generally associated with an increased risk for adverse health outcomes for both mother and child, including high birth weight, cesarean section, shoulder dystocia, preeclampsia, and increased risk of obesity and impaired glucose metabolism in the long-term.^1–3^ GDM treatment usually consists of frequent glucose measurements and lifestyle and dietary changes; a minority of women with GDM are additionally treated with insulin and antidiabetic medication.^1^ A recent systematic review to inform the US Preventive Services Task Force (USPSTF) found a significant association between GDM treatment and improved pregnancy outcomes.^4^ To detect asymptomatic GDM, initiate treatment, and prevent the associated adverse pregnancy outcomes, many countries have established GDM screening programs based on an oral glucose tolerance test (oGTT) at the end of the second pregnancy trimester.^1^

In Norway, the risk factors determining eligibility for GDM screening were revised in 2017 to include a broader target population.^5,6^ The current screening guidelines target women who are nulliparous and 25 y or older, multiparous and 40 y or older, have a body mass index (BMI) of 25 kg/m^2^ or more, or are considered at risk due to their ethnic or familial background or previous pregnancy complications, which includes most pregnant women. There is an ongoing discussion of screening eligibility criteria, however, about whether to use more restrictive criteria through higher age or BMI thresholds or offer the screening test to all women since GDM is prevalent among all age and BMI groups.^7^

Conversely, a systematic Cochrane Review on the direct effects of GDM screening from 2017 expressed uncertainty about the health benefits for mothers and their children resulting from a GDM screening strategy targeting all pregnant women.^8^ According to priority-setting criteria in Norway, the cost-effectiveness of new interventions and their uncertainties should be evaluated prior to implementation.^9^ Decision-analytic models that synthesize different sources of evidence with quantitative methods can assist decision makers in their choice on the allocation of health care resources,^10^ for instance in this case of limited direct clinical and empirical evidence on the cost-effectiveness of GDM screening.^4^

A recent systematic review of cost-effectiveness analyses of GDM screening found that broader screening is more likely to be cost-effective than screening based on risk factors or no screening.^11^ Importantly, the included studies differed in their assumptions about the impact of screening on preventing long-term health effects, reflecting inconsistent and sparse evidence related to long-term outcomes.^12^ For instance, systematic reviews from Cochrane^13^ and USPSTF^12^ reported relative risk (RR) values for the effectiveness of GDM treatment associated with later maternal type 2 diabetes mellitus (T2DM) that are not statistically significant but differ in directionality. Among the 6 studies focusing on prenatal GDM management in another systematic review of economic evaluations of prenatal and postpartum interventions, findings about the cost-effectiveness were inconclusive.^14^ The authors of this latter review also pointed out the scarcity of trial or observational data necessary to better determine the cost-effectiveness of postpartum interventions to reduce the risk of, for example, later T2DM.

Despite high parameter uncertainty, particularly related to long-term effects, only one^15^ of the previous studies included a value-of-information (VOI) analysis, which enables quantification of decision uncertainty and the value of collecting additional evidence to reduce parameter uncertainty through conducting additional research.^16^ Even if a model might not describe a specific policy decision, VOI analysis can be used as a sensitivity analysis to detect those model parameters that contribute most to uncertainty.^17^ Moreover, which outcomes to include in a model-based cost-effectiveness analysis represents a type of structural uncertainty, which should be assessed as part of the uncertainty assessment.^10,18^

Our objective was to use a decision-analytic model to explore the impact of including uncertain, and often conflicting, long-term outcomes on the cost-effectiveness of alternative screening strategies for GDM. We conducted a VOI analysis to identify those parameters with the highest impact on decision uncertainty.^16^

## Methods

### Analytic Overview

We conducted a model-based cost-effectiveness analysis to compare the health and economic consequences of 4 GDM screening strategies targeting women with singleton pregnancy during pregnancy weeks 24 to 28 in Norway without prior diabetes mellitus type 1 or 2, which would be excluded from GDM screening. We conducted primary, secondary, and scenario analyses to assess structural uncertainty related to inclusion and exclusion of certain events. In our primary analysis, we included short-term maternal outcomes related to GDM screening (i.e., outcomes during pregnancy and the postpartum period). Given the short-term time horizon, we developed a probabilistic decision tree. We varied influential parameters and structural assumptions in scenario analysis (see Appendix Tables A-1, A-5.1, and A-5.2). In the secondary analysis, we considered the impact of inconclusive evidence on the preventive effect of GDM treatment on developing T2DM later in life, informed by 2 different sources.^12,13^ In both the primary and secondary analyses, we included the probability of perinatal death and the associated loss of QALYs over a lifetime horizon for a healthy newborn and assumed GDM screening had a preventive effect.

To parameterize model inputs, we used data from the Medical Birth Registry of Norway, meta-analyses, other published literature, clinical guidelines and estimations from the Norwegian Directorate for Health, and Norwegian hospital and general practitioner reimbursement tariffs. We defined our probability parameters as beta, cost and utility parameters as gamma, and RR parameters as lognormal distributions following the suggestions from Briggs et al.^19^ and assuming the standard error to be 20% of the mean value if no information on parameter uncertainty was reported.

We calculated model outcomes for a cohort of 50,000 pregnant women and their offspring, which roughly reflects the current number of births per year in Norway (56,060 births in 2021).^20^ The main model outcomes were total costs and total quality-adjusted life-years (QALYs) per screening strategy. We reported results as the means of outcomes across 10,000 probabilistic simulations. By ranking the strategies from lowest to highest total costs and removing dominated strategies, we calculated the incremental cost-effectiveness ratio (ICER). We determined cost-effectiveness assuming the commonly cited Norwegian benchmark for cost-effectiveness of $28,400 ($1 = 9.675 NOK purchasing power parities).^21,22^ In line with Norwegian guidelines,^23^ we adopted an extended health care perspective (i.e., including time and travel costs associated with screening and treatment but excluding productivity losses) and discounted costs and benefits by 4% per year. We summarized results in a cost-effectiveness acceptability curve (CEAC) that plots the proportion of cost-effective simulations conditional on the cost-effectiveness threshold.

We explored decision uncertainty using VOI analysis based on the results from probabilistic analysis for both the primary and secondary analyses. We calculated the expected value of perfect information (EVPI) to quantify the total decision uncertainty inherent in the model and used the Sheffield Accelerated Value of Information online application^24^ to estimate the expected value of partial perfect information (EVPPI) for individual parameters. To address the potential implications of each screening policy over the mid-term future, we calculated both population EVPI and EVPPI over a 10-y period, again using the annual discount rate of 4%.

### Screening Strategies

We assumed women were diagnosed with GDM by the 1-step oGTT and treated following the diagnostic criteria and treatment guidelines currently recommended in Norway.^6^ The treatment guidelines include dietary and lifestyle advice, self-measurement of blood glucose levels, and referral to specialist health care services where treatment with insulin or metformin is evaluated, if blood glucose levels do not normalize. We did not consider the measurement of HbA1c in the first trimester to detect overt diabetes or early GDM^6^ and excluded women with multiple births or preexisting diabetes from this analysis due to their frequent exclusion in clinical trials.

We analyzed 4 screening strategies that cover the range of possible eligibility criteria: 1) universal screening, 2) current screening guidelines in Norway, 3) high-risk screening, and 4) no screening (Table 1). Universal screening assumes that all pregnant women are eligible for the oGTT. The current screening strategy reflects the current Norwegian eligibility criteria (see the introduction).^6^ Finally, in the high-risk screening strategy, only women fulfilling the criteria that have been associated with a high risk for GDM were considered eligible for screening, which includes age 40 y or older, ethnic background, previous GDM, or previous preeclampsia.^1,25^

**Table 1:** Overview of Screening Eligibility Criteria, Eligibility, and Prevalence Rate per Screening Strategy

	Universal Screening	Current Screening	High-Risk Screening	No Screening
Screening eligibility criteria	All pregnant women	≥25 y and nulliparous or ≥40 y and multiparous or ≥25.0 kg/m^2^ prepregnancy BMI or ethnic background from Asia/Africa or first-degree relative with diabetes or GDM in a previous pregnancy or preeclampsia in a previous pregnancy	≥40 y or ethnic background from Asia/Africa or GDM in a previous pregnancy or preeclampsia in previous pregnancy	No pregnant women
% of women eligible	100.0	73.1^27^	19.2^27^	0.0
GDM prevalence in eligible women in %^a^	10.3^7^	11.6^7^	27.0^7^	—

BMI, body mass index; GDM, gestational diabetes mellitus.

a We assumed the GDM prevalence in the high-risk screening group was similar to the prevalence reported in our source for the study population with a non-European background, as women with a non-European background represented the majority of the eligible women in the high-risk screening group. We further assumed that the reported criteria of multiparity combined with maternal age over 35 y in our source (5 y lower than the actual screening criteria in our model) represented a sufficient approximation for the prevalence related to this criteria in the current screening group in our analysis.

### Model Structure and Disease Outcomes

We selected a decision tree model structure to reflect each of the screening pathways and developed the model in Microsoft Excel version 2204. We structured the decision tree model into 2 linked modules: a screening module and a disease outcome module. The screening module (Appendix Figure A-1) determined the proportion of the cohort in 1 of 4 treatment categories: (A) normal glucose tolerance, (B) undiagnosed and hence untreated GDM, (C) GDM with lifestyle treatment, and (D) GDM with pharmacologic treatment (insulin or metformin). This proportion was conditional on the following parameters: the number of women eligible for screening per screening strategy, screening adherence, screening test characteristics, GDM prevalence per screening strategy, and treatment adherence. Since we based our treatment effect estimates on clinical trials following the intention-to-treat principle, we assumed perfect treatment adherence. Consequently, untreated GDM always refers to undiagnosed GDM in this analysis. We also assumed perfect screening adherence but varied this assumption in scenario analysis.

**Figure 1:**
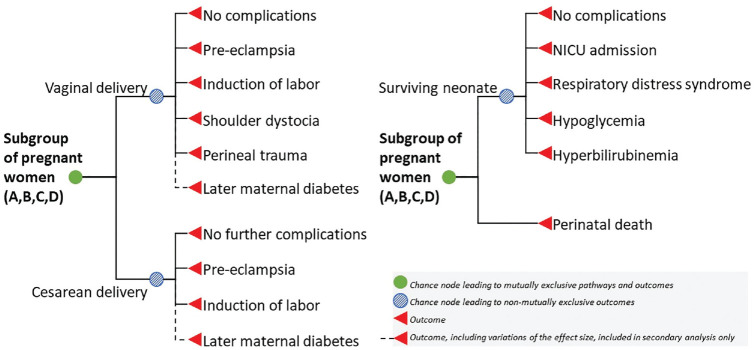
Model schematic for the disease outcome module. Schematic showing the disease outcomes modeled with the probabilistic decision tree model. The probability of disease outcomes was dependent on the subgroup of pregnant women with either normal glucose tolerance (A), untreated GDM (B), lifestyle GDM treatment (C), or pharmacologic GDM treatment (D). The outcome later maternal diabetes type 2 was included in the secondary analysis only, using 2 different relative risk rates reported in systematic reviews on the effect of GDM treatment. GDM, gestational diabetes mellitus; NICU, neonatal intensive care unit.

The disease outcome module projected the adverse pregnancy outcomes conditional on the 4 treatment categories (Figure 1). We included outcomes defined for treatment effect in the USPSTF systematic review, comprising cesarean section, preeclampsia, induction of labor, shoulder dystocia, perineal trauma, admission to neonatal intensive care unit or neonatology, and 3 short-term neonatal complications.^4^ Analogous to some of our data sources, we did not distinguish between emergency and planned cesarean section. We assumed shoulder dystocia and perineal trauma to occur only in vaginal delivery^15^ and all other complications to be independent of the mode of delivery. By basing our model parameters for treatment effectiveness on the results of clinical trials, we implicitly accounted for any interdependencies between outcomes and the potentially higher propensity of clinicians to perform cesarean deliveries and labor induction in women with known GDM.^12^ In addition, we included perinatal death as a disease outcome due to its severe yet rare occurrence^13^ but omitted this outcome in one of our scenarios in the scenario analysis. Since preterm delivery and macrosomia do not directly affect QALY values or health care costs, we did not consider these intermediate outcomes. In the secondary analysis, we also included the outcome T2DM.

We validated the model using the TECH-VER verification checklist.^26^ We also compared model parameters and results to the rates of adverse pregnancy outcomes from the Medical Birth Registry of Norway that were not used in model development (Appendix 6).

### Epidemiologic and Effect Data

In the screening module, the proportion of women fulfilling eligibility criteria was informed by aggregated data from the Medical Birth Registry of Norway,^27^ which covers all maternity units in Norway (Table 1).^28^ Family history of diabetes as an additional risk factor was informed by a recent study of 4 cohorts of pregnant women in Norway, which we also used to derive the parameters for GDM prevalence dependent on maternal characteristics (Table 1; further details are reported in Appendix Tables A-2.1 to A-2.3).^7^

For the disease outcome module, we defined the probabilities for adverse pregnancy outcomes per GDM treatment category (Table 2). The base-case probabilities among women with normal glucose tolerance were informed by pooling estimates from 3 European cohort or registry studies.^29–32^ Due to a lack of literature values, we used the proportion of women with perineal trauma (sphincter rupture grades 3 and 4) from the Medical Birth Registry in our model, despite potential distortion by cases of undetected GDM and potential underreporting.^33^ We multiplied the base-case probabilities first with the RR of adverse pregnancy outcomes associated with GDM, then with the RR rates associated with GDM treatment, as reported by the USPSTF systematic review from the year 2021.^12^ For our secondary analysis, which considers the additional impact of screening on preventing T2DM, we identified 2 systematic reviews reporting RR rates that differ in directionality. While the USPSTF analysis reported an RR of T2DM for women with treated GDM versus untreated GDM of 1.09 (95% confidence interval [CI]: 0.59 to 2.01), a Cochrane systematic review from 2017 reported a RR of 0.98 (CI: 0.54 to 1.76)^13^; therefore, we varied this assumption within our secondary analysis.

**Table 2:** Overview of Probability Values for Adverse Pregnancy Outcomes per GDM Treatment Category

	Normal Glucose Tolerance (A)	Untreated GDM (B)^a^	Treated GDM (C + D)^a^
Cesarean section	402/2,692 | 14.9%^31^	RR 1.2 [1.05–1.38] | 17.9%^12^	RR 0.95 [0.83–1.08] | 17.0%^12^
Preeclampsia	217/7,299 | 3.0%^29,32^	RR 1.93 [1.34–2.77] | 5.7%^12^	RR 0.60 [0.35–1.01] | 3.4%^12^
Induction of labor	1,207/5,543 | 21.8%^31,32^	RR 1.13 [0.93–1.39] | 24.6%^12^	RR 1.18 [0.92–1.52] | 29.0%^12^
Perineal trauma (sphincter rupture grade 3–4)	2,034/150,468 | 1.4%^27^	RR 1.19 [0.81–1.76] | 1.6%^12^	RR 1.04 [0.92–1.18] | 1.7%^12^
Shoulder dystocia	57/4,662 | 1.2%^29^	RR 1.79 [1.02–3.15] | 2.2%^12^	RR 0.42 [0.23–0.77] | 0.9%^12^
Admission to NICU or neonatology	734/7,505 | 9.8%^29,32^	RR 1.17 [0.99–1.38] | 11.4%^12^	RR 0.73 [0.53–0.99] | 8.3%^12^
Neonatal respiratory distress syndrome	86/4,662 | 1.8%^29^	RR 0.65 [0.18–2.35] |1.2%^12^	RR 1.05 [0.48–2.28] | 1.3%^12^
Neonatal hypoglycemia	28/4,662 | 0.6%^29^	RR 2.51 [1.72–3.68] |1.5%^12^	RR 1.10 [0.83–1.45 | 1.7%^12^
Neonatal hyperbilirubinemia	316/4,662 | 6.8%^29^	RR 1.32 [1.13–1.54] | 8.9%^12^	RR 0.84 [0.65–1.08] | 7.5%^12^
Neonatal or fetal death	38/8,200 | 0.5%^30,32^	RR 1.66 [0.93–2.95] | 0.8%^13^	RR 0.09 [0.01–1.7] | 0.1%^13^
Later maternal diabetes^b^	63/3,946 | 1.6%^3^	71/663 | 10.7%^3^	USTPF RR 1.09 [0.59–2.01] | 11.7%^12^ Cochrane RR 0.98 [0.54–1.76] | 10.5%^13^

GDM, gestational diabetes mellitus; NICU, neonatal intensive care unit; RR, relative risk.

a We defined all RR rates in our model as lognormal distributions and corrected for the effect of the skewed distribution using the method by Barendregt^49^ to ensure that the mean RR in the Monte Carlo simulation was approximately equal to the RR from the epidemiological literature.

b We included the outcome “later maternal diabetes” in the secondary analysis only.

### Cost and Health-Related Quality-of-Life Assumptions

We calculated the associated costs for each pregnancy outcome and applied a comprehensive estimate of the antenatal and postnatal care costs regardless of GDM diagnosis and treatment status. We based the cost parameters on Norwegian hospital and general practitioner reimbursement tariffs,^34–36^ Norwegian clinical guidelines and reports,^6,37–39^ and a previous health economic analysis,^40^ indexed to price year 2021 using the consumer price index.^41^ For the treatment costs, we assumed that 35% of women diagnosed with GDM require further treatment review after lifestyle and dietary advice^6^ and that 60% of these women will require pharmacologic treatment, based on estimates from the Norwegian Directorate of Health.^40^ Details on unit costs and single parameters are reported in Appendix Tables A-2.4 to A-2.9.

Due to the inconclusive evidence on the impact of GDM screening, diagnosis, and treatment on health-related quality of life,^12,42^ we did not assume any disutility associated with these events. We conducted a structured literature review to identify health state utility values for each adverse pregnancy outcome (see details in Appendix Table A-3.2). Generally, we calculated a QALY loss as the difference between the health state utility of 1.0 for perfect health and the utility value from the literature, multiplied by the assumed duration of the adverse pregnancy outcome (Appendix Table A-3.1). For the long-term outcomes, we based our calculation of QALY loss on the life expectancy from Statistics Norway^43^ and the utility values for the general population from the Norwegian Institute of Public Health (Appendix Table A-4.1 to A-4.3).^23^ For T2DM, we calculated total diabetes-related costs over the expected time frame with T2DM and the total QALY decrement, using fixed parameters for life expectancy and diabetes onset.

## Results

### Primary Analysis

For a cohort of 50,000 women, the model projected that nearly 90% of women (44,829 women) would have normal glucose tolerance across all screening strategies. Universal screening resulted in the highest number of women being treated for GDM (9.8%) compared with the other strategies (8.1% with current screening and 4.9% with high-risk screening) and, conversely, the lowest number of cases of undiagnosed and hence untreated GDM (0.5%, 2.3%, 5.4%, and 10.3% of pregnant women for universal, current, high-risk, and no screening, respectively).

**Figure 2:**
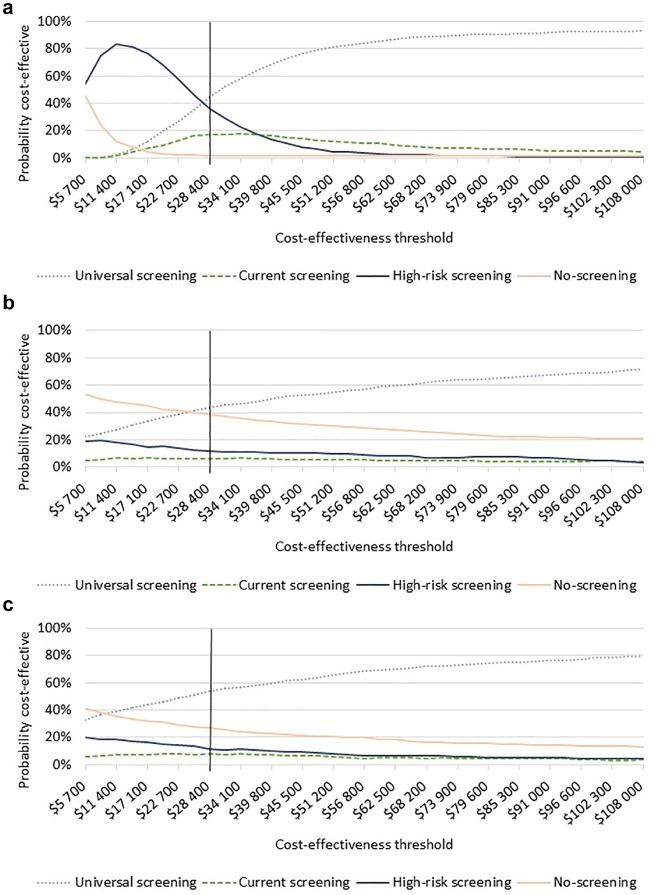
Cost-effectiveness acceptability curves: (a) primary analysis, (b) secondary analysis (USPSTF 2021 RR 1.09 [0.59–2.01]), and (c) secondary analysis (Cochrane 2017 RR 0.98 [0.54–1.76]). Cost-effectiveness acceptability curves showing the percentage of 10,000 simulations from the probabilistic sensitivity analysis, represented along the *y*-axis, considered cost-effective under a given cost-effectiveness threshold, represented along the *x*-axis. A Norwegian benchmark for cost-effectiveness, 28,400 USD, is indicated with a vertical line. Panel (a) shows results from the primary analysis excluding the outcome of later maternal diabetes. Panels (b) and (c) show the results from the secondary analysis including the outcome of later maternal diabetes with 2 different relative risk rates associated with GDM treatment compared with no GDM treatment, using the values reported in USPSTF and Cochrane systematic reviews. The reported 95% confidence intervals for the relative risk values are indicated in brackets. GDM, gestational diabetes mellitus; RR, relative risk; USPSTF, United States Preventive Services Task Force.

In our cost-effectiveness analysis, current screening was not considered an efficient strategy (i.e., extendedly dominated) and was thus removed from further consideration (Table 3). Given a cost-effectiveness threshold of $28,400, universal screening was considered the most cost-effective strategy (ICER: $26,014 per QALY gained), although decision uncertainty remained high. Universal screening and high-risk screening were considered cost-effective in approximately 45% and 36% of the individual probabilistic simulations at a threshold of $28,400, respectively (Figure 2a). However, at higher thresholds (e.g., $50,000), universal screening was considered cost-effective in >80% of the simulations.

**Table 3:** Probabilistic Model Results in the Primary and Secondary Analyses

	Total Discounted Costs	Total Discounted QALY Loss	Δ Total Discounted Costs	Δ Total Discounted QALYs (Gain)	ICER (Cost per QALY Gained)
Primary analysis					
No screening	$636,504,394	−7,041			
High-risk screening	$638,570,828	−6,650	$2,066,434	392	$5,277
Current screening	$645,328,649	−6,400	$6,757,821	250	Extended dominance
Universal screening	$648,679,839	−6,261	$3,351,190	139	**$26,014**
Secondary analysis with RR from USPSTF (Pillay et al. 2021)^12^					
No screening	$883,332,662	−9,529			
High-risk screening	$889,952,488	−9,182	$6,619,826	346	**$19,115**
Current screening	$899,635,509	−8,960	$9,683,021	223	Extended dominance
Universal screening	$904,566,190	−8,838	$4,930,681	122	$42,423
Secondary analysis with RR from Cochrane (Brown et al. 2017)^13^					
No screening	$882,451,154	−9,513			
High-risk screening	$883,480,190	−9,113	$1,029,036	400	$2,571
Current screening	$889,607,089	−8,858	$6,126,899	256	Extended dominance
Universal screening	$892,614,989	−8,716	$3,007,900	142	**$22,997**

ICER in bold marks the most cost-effective strategy under a cost-effectiveness threshold of $28,400.

### Secondary Analysis

When we included the potential preventive effect of screening on later T2DM from 2 alternative sources, universal screening remained cost-effective when we used the preventive effect from the 2017 Cochrane Review (ICER: $22,997 per QALY gained; Table 3). Under this assumption, universal screening was considered cost-effective in more than 50% of the simulations at the threshold of $28,400 (Figure 2c). In contrast, when we assumed the RR of 1.09 (95% CI: 0.59 to 2.01), as estimated for the USPSTF, the cost-effective strategy involved restricting screening eligibility to the high-risk criteria (ICER: $19,115 per QALY gained compared with no screening, Table 3), yet it was cost-effective in just more than 10% of the simulations (Figure 2b).

### VOI Analysis

**Figure 3:**
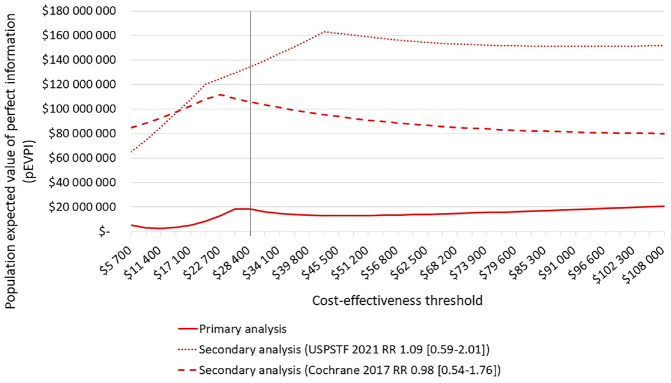
Population expected value of perfect information. Line graph showing the population expected value of perfect information, represented along the *y*-axis, under a given cost-effectiveness threshold, represented along the *x*-axis. A Norwegian benchmark for cost-effectiveness, 28,400 USD, is indicated with a vertical line. The pEVPI was calculated for a cohort of 50,000 women over 10 y, discounted by 4%. The solid line represents results from the primary analysis, excluding the outcome of later maternal diabetes. The dashed lines represent results from the secondary analysis including the outcome of later maternal diabetes with 2 different relative risk rates associated with GDM treatment compared with no GDM treatment, using the values reported in USPSTF and Cochrane systematic reviews. The reported 95% confidence intervals for the relative risk values are indicated in brackets. GDM, gestational diabetes mellitus; pEVPI, population expected value of perfect information; RR, relative risk; USPSTF, United States Preventive Services Task Force.

In the primary analysis, the population EVPI over a 10-y time horizon was less than $20 million, while in both of our secondary analyses, the value of eliminating all parameter uncertainty increased to more than $100 million at the threshold of $28,400 (Figure 3). When we estimated the population EVPPI in the primary analysis, we found that the RR of perinatal death associated with GDM versus normal glucose tolerance, and with treated GDM versus untreated GDM, accounted for most of the value of additional research (i.e., $7.4 million and $6.8 million, respectively). In the secondary analysis and irrespective of applying the Cochrane or USPSTF source, the highest population EVPPI was estimated for the RR of T2DM associated with treated GDM versus untreated GDM. For example, when we assumed an RR of 1.09 (95% CI: 0.59 to 2.01), the population EVPPI was $124.4 million, and when we assumed an RR of 0.98 (95% CI: 0.54 to 1.76), the population EVPPI was $98.6 million. The probability parameters for perinatal mortality continued to contribute substantially to the decision uncertainty but at a lower value than the RR of T2DM.

### Scenario Analysis

In the primary analysis, the most cost-effective screening strategy varied across scenario analyses (Appendix Table A-5.3). When we excluded perinatal mortality from our analysis, GDM screening would not be considered cost-effective irrespective of eligibility criteria. High-risk screening was cost-effective when we assumed a lower risk of adverse pregnancy outcomes (using the lower bound of the 95% confidence interval for RR values) and when we assumed an alternative set of probability parameters leading to a lower probability of perinatal death.

## Discussion

Our model projected that the preferred screening strategy, ranging from high-risk to universal screening, depended on the assumption and inclusion of a preventive effect of GDM treatment on T2DM. In addition, the value of any screening irrespective of eligibility criteria was dependent on the effects of GDM treatment on preventing perinatal death. It should be noted, however, that even with a “no-screening strategy”, we would expect that clinicians will initiate some form of screening for women considered at very high risk of GDM. Norway’s current screening guidelines were consistently cost-inefficient in all analyses. As the RR of the 2 outcomes perinatal death and T2DM contributed most to decision uncertainty according to our EVPPI results, and their inclusion in the model represents an important source of structural uncertainty, they require further discussion.

First, improving perinatal health outcomes, especially averting perinatal deaths, may trigger a special status within priority setting (e.g., warranting a higher cost-effectiveness threshold or a higher acceptance for uncertainty). High survival rates for preterm infants due to high-quality antenatal and neonatal care in Norway^38^ indicate that averting perinatal deaths is a high priority in the Norwegian health care system. This consideration is in line with the Norwegian Health Directorate referring to GDM treatment as an intervention to reduce clinically important complications for both mother and child.^6^ Our finding that the cost-effectiveness of GDM screening depends largely on the impact on perinatal mortality supports this consideration.

As infants born to mothers with GDM have a higher risk of being macrosomic, they have a higher risk of other severe complications such as birth injuries, asphyxia, bone fractures, and nerve palsies,^13^ which we did not account for in the current analysis due to lacking data. Importantly, these complications can result in lifelong disabilities with an impact on the quality of life of the affected children and their parents and economic impacts such as health care costs and lost productivity. However, incorporating these potential joint effects into cost-effectiveness analyses remains a methodological challenge. Furthermore, we did not account for the impact of perinatal death on the parents’ quality of life beyond the postpartum period; that is, the loss of a child will likely affect their mental health over their lifetime. If GDM screening prevented these additional outcomes, universal screening would remain the preferred strategy.

Second, when we included T2DM as an outcome, the preferred strategy was either universal or high-risk screening, depending on the source of the evidence, and in both cases, decision uncertainty increased. From a methodological perspective, the high parameter uncertainty of a few parameters translated into high expected value of perfect and partial perfect information, which suggests a high value of collecting additional evidence to reduce parameter uncertainty. The research design of future studies on the GDM treatment effect, however, is challenging. Lifestyle interventions and GDM treatment provide benefits to mother and child^13^ that cannot be randomly withheld from a control group in a clinical trial. Lifestyle advice on physical exercise and healthy eating is routinely offered to all pregnant women in Norway within antenatal care, which would make it challenging to identify the treatment effect from an additional GDM-specific lifestyle intervention.^39^ In addition, by knowing about their GDM diagnosis, women and their health care specialists might monitor the pregnancy more closely, and thus, the treatment effect may partly stem from knowing rather than from active treatment. For instance, the authors of one large GDM treatment trial suggested that bias due to increased awareness might be present in their results, even though they refuted a severe bias based on the plausibility of their results.^44^ Despite these challenges, our findings motivate, for example, additional follow-up studies that could help to determine the prevention effect of GDM treatment on later metabolic distortions for both pregnant women and their children. Currently, most postpartum follow-up studies are between 4 and 10 y long, and the results from longer studies are not yet available.^1^ Future research could also provide more information on how to feasibly collect more evidence and how to incorporate follow-up monitoring of T2DM in a comprehensive model of the cost-effectiveness of preventive treatment for T2DM.

Follow-up is also an important aspect with regard to the monitoring of T2DM. In our model, we included the costs for 1 medical appointment with HbA1c measurement 4 mo postpartum as per the Norwegian GDM guidelines.^6^ However, we did not consider other possibilities for the prevention or earlier detection of T2DM, for instance, postpartum lifestyle advice or monitoring of postpartum weight change. Post-GDM follow-up will presumably lead to additional costs related to health care appointments but may result in long-term health benefits. Since GDM is associated with a higher risk of disorders in glucose metabolism,^3^ as well as hypertension and ischemic heart disease,^45^ identifying women with GDM may help identify women at increased risk of T2DM and other noncommunicable diseases. In turn, identifying these women and providing health care services may provide additional benefits not captured in our analysis. Furthermore, we did not account for the possibility that GDM treatment during or after pregnancy may delay the onset of T2DM.

We restricted our analysis to 4 screening strategies. While alternative combinations of eligibility criteria along the cost-efficient frontier in between high-risk and universal screening are imaginable, we do not expect that our cost-effectiveness results would drastically deviate when comparing more alternative strategies. In the most recent study on GDM prevalence with varying maternal characteristics in Norway,^7^ the most effective strategies in terms of identifying the most women with GDM (i.e., more than 80%), were close to universal screening. Nevertheless, in the scenarios in which we found high-risk screening to be the most cost-effective strategy, including and thus detecting just a few more women with GDM might still be considered cost-effective. For instance, while there is no clear cutoff BMI value that distinguishes women with elevated GDM risk,^25^ obesity could be an additional criterion to define screening eligibility. Finding the optimal screening algorithm continues to be debated, with the current screening criteria not leading to a significant difference in GDM prevalence, as shown in a recent study from Northern Norway.^5^

When comparing the model results of the proportion of adverse pregnancy outcomes with data from the Medical Birth Registry, our model predicted results for the current screening with less than 1 percentage point deviation, except for the outcome of induction of labor (see Appendix 6). When comparing the parameter values for the probability of adverse pregnancy outcomes with treated GDM with data from the registry, however, the model appeared to underestimate most risk rates. We hypothesized that the RR rates used in the model are not perfectly applicable to the Norwegian diagnostic thresholds for GDM, which are higher than the commonly used IADPSG criteria.^6,46^ For some outcomes, the probability of adverse pregnancy outcomes was lower with treated GDM compared with normal glucose tolerance. Since the birth from a pregnancy complicated by GDM is monitored more closely, this paradox could be correct. Nevertheless, it raises the general question about how valid the assumed treatment effect is and whether it can be extrapolated to all women, if more than just the high-risk population was screened.

Future cost-effectiveness models should monitor the increasing prevalence of GDM,^47^ which will lead to a higher proportion of women detected in broad screening strategies. Finally, the uncertainty in risk rates and treatment effects in women diagnosed with the Norwegian diagnostic criteria and the ongoing debate about the optimal diagnostic threshold values^48^ could be addressed with more research on the association between diagnostic thresholds and adverse pregnancy outcomes using individual patient-level data.

## Conclusions

Our model-based analysis revealed large uncertainty related to the cost-effectiveness of GDM screening. We found that Norway’s current screening recommendations were considered inefficient under all analyses, while universal screening for GDM was considered cost-effective, provided that screening has a preventive effect on perinatal mortality. Less favorable assumptions about potential benefits on T2DM, however, would support restricting the screening policy to the high-risk population. We also showed that the decision uncertainty on the most cost-effective screening strategy was concentrated to a few parameters, namely, T2DM and the risk of perinatal death associated with GDM and GDM treatment. With these findings, we add to other studies emphasizing the importance of further research into the long-term effects of GDM screening and treatment for pregnant women and their children. In turn, these studies can help inform the design of future screening guidelines and shed light on the underlying balance between benefits and harms of GDM screening.

## Supplemental Material

sj-pdf-1-mdm-10.1177_0272989X241241339 – Supplemental material for Exploring Structural Uncertainty in Cost-Effectiveness Modeling of Gestational Diabetes Screening: An Application Example from Norway Supplemental material, sj-pdf-1-mdm-10.1177_0272989X241241339 for Exploring Structural Uncertainty in Cost-Effectiveness Modeling of Gestational Diabetes Screening: An Application Example from Norway by Pia S. Henkel, Emily A. Burger, Line Sletner and Kine Pedersen in Medical Decision Making
