# Supplementary material for: Exploring Structural Uncertainty in Cost-Effectiveness Modeling of Gestational Diabetes Screening: An Application Example from Norway
Source: Med Decis Making. 2024 Apr 9;44(4):380–92. doi: 10.1177/0272989X241241339 (PMC11102644; doi:10.1177/0272989X241241339)
Supplement: sj-pdf-1-mdm-10.1177_0272989X241241339 – Supplemental material for Exploring Structural Uncertainty in Cost-Effectiveness Modeling of Gestational Diabetes Screening: An Application Example from Norway [file sj-pdf-1-mdm-10.1177_0272989X241241339.pdf]

# Appendix List of content


## Appendix 1 Analytic overview

Figure A-1: Screening module schematic
Table A-1: Overview of disease outcomes included in primary, scenario and secondary analysis

## Appendix 2 Parameter details

Table A-2.1 Probability parameters for the screening module
Table A-2.2 Calculation of screening eligibility
Table A-2.3 Calculation of GDM prevalence based on reported diagnostic accuracy values
Table A-2.4 Composite cost parameters for GDM screening and treatment
Table A-2.5 Composite cost parameters for pregnancy outcomes associated with GDM
Table A-2.6 Composite cost parameters for general antenatal and postnatal care costs
Table A-2.7 Unit costs for the calculation of composite cost parameters
Table A-2.8 Proportion of births with and without complications for vaginal delivery and cesarean section
Table A-2.9 Calculation of weighted average reimbursement weight for pre-term birth in pre-eclampsia cases

## Appendix 3 Short-term QALY values

Table A-3.1 Calculation of QALY decrements from short-term pregnancy outcomes
Table A-3.2 Structured literature review for short-term QALY values

## Appendix 4 Calculation of long-term costs and QALY loss

Table A-4.1 Parameters for the calculation of long-term costs and QALY loss
Table A-4.2 Calculation of lifetime costs and QALY loss from later maternal diabetes type 2
Table A-4.3 Calculation of lifetime QALY loss from perinatal death

## Appendix 5 Scenario analysis

Table A-5.1 Scenarios in scenario analysis
Table A-5.2 Parameter details for scenario 12 including large for gestational age
Table A-5.3 Probabilistic model results in scenario analysis

## Appendix 6 Model validation

Table A-6.1 Validation of probability parameters for adverse pregnancy outcomes with treated GDM
Table A-6.2 Validation of model results in the current screening strategy with Norwegian registry data

## Appendix 7 CHEERS reporting checklist

## References

# Appendix 1 Analytic overview

Figure A-1 Screening module schematic

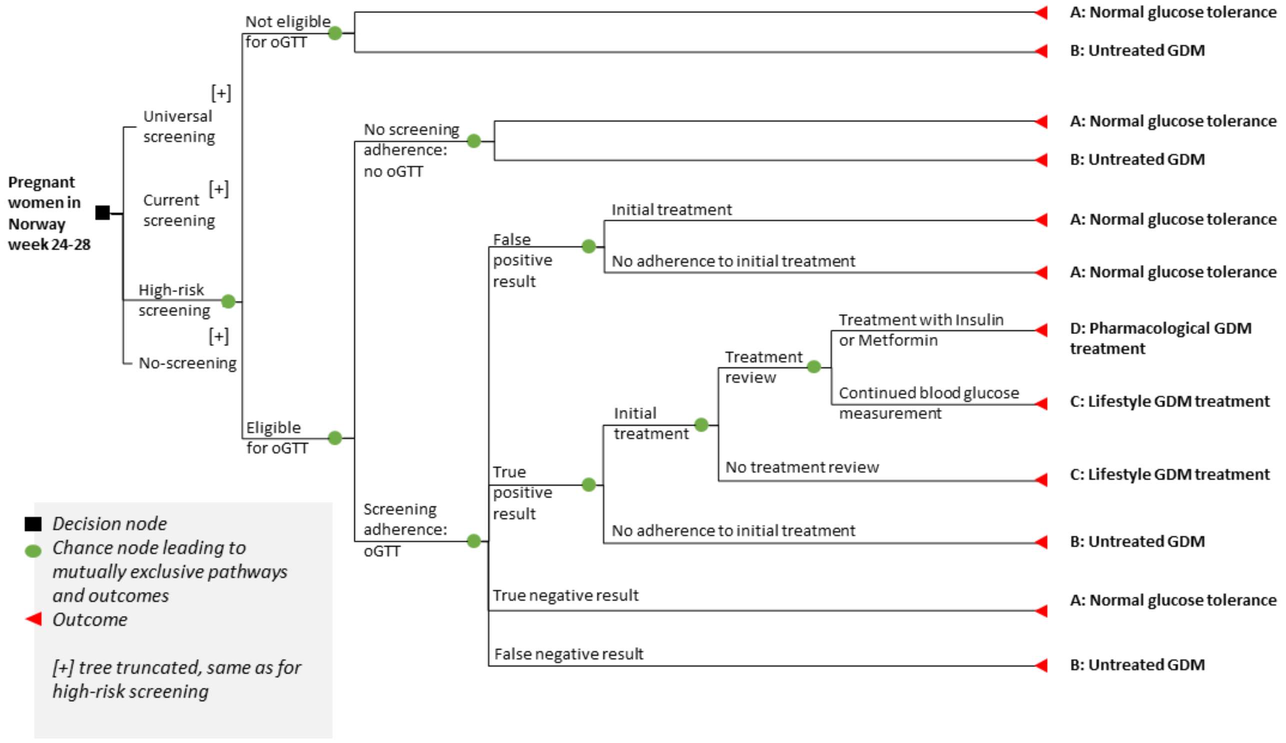

Table A-1 Overview of disease outcomes included in primary, scenario and secondary analysis

| | **Primary analysis** | **Scenario analysis** | **Secondary analysis** |
|---|---|---|---|
| Cesarean section | ✓ | ✓ | ✓ |
| Preeclampsia | ✓ | ✓ | ✓ |
| Induction of labor | ✓ | ✓ | ✓ |
| Perineal trauma (sphincter rupture grade 3-4) | ✓ | ✓ | ✓ |
| Shoulder dystocia | ✓ | ✓ | ✓ |
| Admission to NICU or neonatology | ✓ | ✓ | ✓ |
| Neonatal respiratory distress syndrome | ✓ | ✓ | ✓ |
| Neonatal hypoglycemia | ✓ | ✓ | ✓ |
| Neonatal hyperbilirubinemia | ✓ | ✓ | ✓ |
| Neonatal or fetal death | ✓ | Scenario 1-12: ✓<br>Scenario 13: ✗ | ✓ |
| Later maternal diabetes | ✗ | ✗ | ✓<br>Cochrane or USPSTF |
| Large for gestational age | ✗ | Scenario 1-11, 13: ✗<br>Scenario 12: ✓ | ✗ |

## Appendix 2 Parameter details

Table A-2.1 Probability parameters for the screening module

| Parameter | Value | Source |
|---|---|---|
| Screening eligibility universal screening | 100% | Assumption |
| Screening eligibility high-risk screening | 19.2% | Composite parameter, see Table A-2.2 for details |
| Screening eligibility current screening | 73.1% | Composite parameter, see Table A-2.2 for details |
| Screening eligibility no-screening | 0% | Assumption |
| Screening adherence all strategies | 100% | Assumption |
| GDM prevalence in general population | 308/2981 \| 10.3% | [1] |
| GDM prevalence for current screening | 234/2012 \| 11.6% | Estimation based on [1], see Table A-2.3 for details |
| GDM prevalence for high-risk screening | 106/393 \| 27.0% | Estimation based on [1], see Table A-2.3 for details |
| Sensitivity oGTT | 95.0% | Assumption, same as [2] |
| Specificity oGTT | 95.0% | Assumption, same as [2] |
| Treatment adherence | 100% | Assumption, same as [3] |
| Referral to treatment review with specialist healthcare service | 35.0% (standard error 0.07) | [4] standard error assumed to be 20% of the mean |
| Insulin and metformin treatment after treatment review | 60.0% (standard error 0.12) | [5] standard error assumed to be 20% of the mean |

Table A-2.2 Calculation of screening eligibility

| Characteristic | Absolute proportion | Relative proportion |
|---|---|---|
| 40+ age, born in an Asian or African country[a] or previous preeclampsia | 8,735 of 51,810 births in 2020 | 16.9% |
| Parity 1+ | 29,537 out of 51,810 births in 2020 | 57.0% |
| GDM diagnosis | 13,811 out of 277,823 births in 2015-2019 | 5.0% |
| Previous GDM | Calculated from above | 2.8% |
| 40+ age, born in an Asian or African country, previous preeclampsia, or previous GDM | | **19.2%** |
| Parity 0 and 25+ age, parity 1+ and 40+ age or BMI 25+ | 30,330 out of 47,635 births in 2020 (with BMI information) | 63.7% |
| Born in an Asian or African country | 6,943 out of 51,810 births in 2020 | 13.4% |
| Family history of diabetes | 350 out of 2,981 women in cohort study [1] | 11.7% |
| Previous preeclampsia | 183 out of 51,810 births in 2020 | 0.4% |
| Parity 0 and 25+ age, or parity 1+ and 40+ age, or BMI 25+, or born in an Asian or African country, or family history of diabetes, or previous preeclampsia, or previous GDM | | **73.1%** |

**a:** Since the Medical Birth Registry does not record ethnic background, we used the mother's country of birth as a proxy.

**Source:** We defined screening eligibility as composite parameter, calculated based on aggregated data from the Medical Birth Registry[6] and from a study on GDM prevalence in Norway[1] (Table A-2.3). The registry data excluded multiple pregnancies (twins, triplets, etc.) and women with diabetes type 1 or 2. The assumed relative proportions for screening eligibility consider that women can exhibit more than one characteristic that would make them eligible for screening. We accounted for this by deducting the share of women being already covered per new criteria, assuming equal distribution of characteristics across the population. For example, 19.2% was calculated as 16.9%+2.8%-(16.9%*2.8%).

Table A-2.3 Calculation of GDM prevalence based on reported diagnostic accuracy values

| | **European background n=2,588**<br>BMI ≥25 or<br>(Primipara + age≥25) or<br>(parous + age≥40) | **Non-European background n=393**<br>BMI ≥25 or<br>(Primipara + age≥25) or<br>(parous + age≥35) | **Total n=2981** |
|---|---|---|---|
| Reported values | | | |
| Sensitivity | 78.7 | 69.8 | |
| Specificity | 32.3 | 42.5 | |
| Positive predictive value | 9.0 | 31.0 | |
| Negative predictive value | 94.7 | 79.2 | |
| oGTTs needed | 68.5 | 60.8 | |
| Undetected cases | 21.3 | 30.2 | |
| Calculated values | | | |
| n with these criteria | 1,773 | 239 | 2012 |
| GDM cases with these criteria | 160 | 74 | 234 |
| Prevalence with these criteria | 9.0% | 31.0% | 11.6% |
| Total GDM cases in this group | 203 | 106 | 309 |
| Total prevalence in this group | 7.8% | 27.0% | 10.4% |

**Source:** Since the Norwegian cohort study on GDM prevalence[1] stratified by maternal characteristics only reported diagnostic accuracy values, we calculated the underlying number of GDM cases in the cohort group. We used the derived GDM prevalence values (Table A-2.1) as model input.

Table A-2.4 Composite cost parameters for GDM screening and treatment

| Parameter | Cost per woman in 2021 USD[a] from an extended healthcare (healthcare only) perspective | Source | Assumption and Justification |
|---|---|---|---|
| Cost for determining screening eligibility | 0 | Assumption | No additional costs since screening eligibility will be assessed within routine antenatal care |
| Cost for oGTT consultation | 210 (92) | 5,7-9 | Duration is 0.5 hours for the healthcare service and 4 hours for the woman, incl. travel and waiting time<br>40% of women will have oGTT with their GP: standard tariff for 20min consultation + supplement for additional time<br>60% of women will have oGTT with a midwife: cost per hour based on average gross monthly salary incl. fees and social costs<br>Patient time cost per hour (leisure time) is based on average salary in Norway after tax<br>Patient travel costs is based on Medicines agency data on travels without requisition, multiplied with 2 to reflect way there and back<br>50% of women will have oGTT during routine consultation – no additional travel costs<br>Standard tariff for material costs and blood sample laboratory examination |
| Cost for lifestyle advice after GDM diagnosis | 417 (211) | 5 | Two consultations per woman<br>Duration is 1 hour for the healthcare services and 3 hours for the patient<br>Consultation takes place at GP's or midwife's office, costs and proportion covered by midwife as stated before<br>Patient time and travel costs as stated before |
| Cost for treatment review with specialist health services | 272 (169) | 5,10 | Duration is 3 hours for the patient incl. travel and waiting time<br>Cost of specialist care diabetes consultation (DRG code 910A)<br>Patient time and travel costs as stated before |
| Cost for self-measurement of blood glucose levels | 179 (48) | 5,11 | 60 self-measurements<br>Duration per self-measurement is 5 mins<br>Pharmacy refund prices minus 25% value added tax for self-measurement strips<br>Patient time costs as stated before |
| Cost for pharmacological treatment (insulin or metformin) | 42 | 4,12,13 | Assumed duration of treatment is 9 weeks based on oGTT in week 26 and 4 weeks of lifestyle treatment before pharmacological treatment<br>3x500mg tablets of metformin daily for 50% of treated women, 34 units of insulin daily for 50% of women, using pharmacy refund prices minus 25% value added tax |
| Cost for additional ultrasound after GDM diagnosis in week 36 | 238 (135) | 5,10 | Duration is 3 hours for the patient incl. travel and waiting time<br>Cost of fetal diagnostic examination (DRG code 914Q)<br>Patient time and travel costs as stated before |

| | | | |
|---|---|---|---|
| Cost for post-partum follow-up with HbA1c measurement | 140 (63) | 5,8 | Standard tariff for 20min GP consultation and HbA1c measurement<br>Duration is 2 hours for the patient incl. travel and waiting time<br>Patient time and travel costs as stated before |

a: $1 = 9.675 NOK purchasing power parities

Table A-2.5 Composite cost parameters for pregnancy outcomes associated with GDM

| Parameter | Cost per woman in 2021 USD[a] from an extended healthcare (healthcare only) perspective | Source | Assumption and Justification |
|---|---|---|---|
| Cost for vaginal delivery | 4,855 (3,191) | 10,14 | Average cost of vaginal delivery with and without complications (DRG codes 372 and 373)<br>Weighed by proportion of vaginal deliveries with complications as reported by DRG coding in 2018<br>Average length of stay for patient time cost is 2.6 days<br>Patient time cost per hour and travel costs as stated before |
| Cost for cesarean section | 10,367 (7,883) | 10,14 | Average cost of cesarean section with and without complications (DRG codes 370 and 371)<br>Weighed by proportion of cesarean deliveries with complications as reported by DRG coding in 2018<br>Average length of stay for patient time cost is 3.9 days<br>Patient time cost per hour and travel cost as stated before |
| Cost for birth monitoring when GDM is pharmacologically treated | 126 | 10,15 | According to obstetrics guidelines, continuous cardiotocography and STAN/lactate measurement for women with medically treated GDM<br>Procedure code 14O04 in DRG code 914P for obstetric diagnostic measures |
| Cost of preeclampsia in vaginal delivery | 4,306 | 5,10,16,17 | Difference between vaginal delivery with and without complications (DRG codes 372 and 373) plus costs of preterm births for 6.2% (729/11,801) of preeclampsia cases calculated as weighed average of DRG codes for low-weight newborns minus general cost of newborn |
| Cost of preeclampsia in cesarean section | 5,402 | 5,10,16,17 | Difference between cesarean section with and without complications (DRG codes 370 and 371) plus costs of preterm births for 6.2% of preeclampsia cases calculated as weighed average of DRG codes for low-weight newborns minus general cost of newborn |
| Cost of induction of labor | 372 | 10 | DRG code 814R for other delivery related procedure |
| Cost of perineal trauma (sphincter rupture grade 3 or 4) | 1,703 (1,626) | 10,15,18 | Cost of operation for anal sphincter injury (indexed to 2021) requiring treatment in the operating room based on obstetrics guidelines requiring surgery in operating room for perineal tears grade 3 or 4<br>Additional cost of one outpatient consultation regarding postpartum period (DRG code 914O) for follow-up<br>Assumed duration for follow-up is 2 hours<br>Patient time cost per hour and travel costs as stated before |
| Cost of shoulder dystocia | 1,478 | 10 | Difference between vaginal birth with and without complications (DRG codes 372 and 373), assumed to not occur in cesarean section |
| Cost of newborn | 2,540 | 10 | General cost of fresh newborn without any problem (DRG code 391) |
| Cost of NICU admission | 32,176 (28,392) | 10,19 | Difference between cost of pre-term or term-newborn with multiple problems (DRG codes 388A and 389B) and general |

| | | | |
|---|---|---|---|
| | | | newborn (391) and newborn with other significant problem (390)<br>Proportion weighed by 40% of newborns admitted to NICU that are pre-term according to numbers from 2009-2014<br>Time costs for the mother or the other parent based on 12 days average length of stay in NICU, assuming 50% of time lost<br>Time cost per hour stated as before |
| Cost of neonatal respiratory distress syndrome | 2,236 | 10 | Difference between newborn with other significant problem and general newborn (DRG codes 390 and 391) |
| Cost of neonatal hypoglycemia | 2,236 | 10 | Difference between newborn with other significant problem and general newborn (DRG codes 390 and 391) |
| Cost of hyperbilirubinemia | 2,236 | 10 | Difference between newborn with other significant problem and general newborn (DRG codes 390 and 391) |
| Cost of perinatal death | 6,572 | 10 | Newborn death within 0-2 days (DRG code 385A) |

a: $1 = 9.675 NOK purchasing power parities

Table A-2.6 Composite cost parameters for general antenatal and postnatal care costs

| Parameter | Cost per woman in 2021 USD[a] from an extended healthcare (healthcare only) perspective | Source | Assumption and Justification |
|---|---|---|---|
| Cost for antenatal care | 587<br>(258) | 7,8 | Standard tariff for first antenatal check-up and 5 further check-up consultations at the General practitioner<br>Assumed duration is 2 hours for the patient, including travel and waiting time<br>Patient time and travel costs as stated before |
| Cost for postnatal care | 225<br>(172) | 10,20 | 1 home visit by a midwife and a public health nurse each, together 2 home visits<br>Duration of each visit is 1 hour<br>Patient and midwife time costs per hour as stated before<br>Cost of outpatient consultation (DRG code 914O) for the health nurse visit |

a: $1 = 9.675 NOK purchasing power parities

Table A-2.7 Unit costs for the calculation of composite cost parameters

| Unit | Unit value (standard error[a,b]) | Source |
|---|---|---|
| Assumed number of 500mg tablets of metformin needed for treatment, assuming 3 tablets daily for 9 weeks | 189 (37.8) | [4] |
| Pharmacy refund price for package of 100 tablets with 500mg active substance metforminhydrochloride, excl. 25% value added tax | 5.95 USD (1.19) | [12] |
| Total price for 9 weeks treatment with metformin (2 packages) | 11.90 USD | Calculated from above |
| Assumed number of units of insulin needed for treatment, assuming 34 units daily for 9 weeks | 2,142 (428.4) | [4,13] |
| Pharmacy refund price for package of 5x3ml pre-filled pen insulin aspart, protamin, insulin aspart, 100 units/ml, exl. 25% value added tax | 36.04 USD (7.21) | [12] |
| Total price for 9 weeks treatment with Insulin (2 packages) | 72.08 USD | Calculated from above |
| Assumed number of measurement strips needed for self-measurement of blood glucose levels | 60 (12) | [4] |
| Assumed duration of self-measurement per time for the woman | 5 mins (1) | [5] |
| Pharmacy refund price for package of 50 measurement stripc, excl. 25% value added tax | 23.84 USD (4.77) | [11] |
| Total price for test strips needed for self-measurement (2 packages) | 47.68 USD | Calculated from above |
| Standard tariff for 20min consultation at the General Practitioner, code 2ad multiplied by 2 to reflect public subsidy | 34.73 USD (6.95) | [7,8] |
| Supplement tariff for consultation >20mins at the General Practitioner, tariff per started 15mins, code 2cd multiplied by 2 to reflect public subsidy | 45.89 USD (9.18) | [7,8] |
| Average gross monthly salary for a midwife, code 2222 Jordmødre | 6,125 USD | [21] |
| Hourly cost for a midwife based on monthly salary and 1662.5 working hours per year incl. 13% employer's contribution and 25% social costs | 61.01 USD (12.20) | [7] |
| Patient time cost per hour based on average salary in Norway after tax | 26.28 USD (5.26) | [7,21] |
| Patient travel cost way and back based on total travel costs from travel without requisition (patient travels on its own) from health trusts and Helfo, indexed to 2021 using consumer price index | 24.20 USD (4.84) | [7] |
| Standard tariff for blood sample laboratory examination code 701a multiplied by 2 to reflect public subsidy | 13.23 USD (2.65) | [7,8] |
| Standard tariff for material costs code 10c material group 3 multiplied by 2 to reflect public subsidy | 28.53 USD (5.71) | [7,8] |
| Standard tariff for HbA1c measurement code 709 multiplied by 2 to reflect public subsidy | 28.32 USD (5.66) | [7,8] |
| Standard tariff for a first antennal check-up at the general practitioner code 217a multiplied by 2 to reflect public subsidy | 33.07 USD (6.61) | [7,8] |
| Standard tariff for up to 5 further antenatal check-ups at the general practitioner code 217b multiplied by 2 to reflect public subsidy | 18.60 USD (3.72) | [7,8] |
| DRG unit cost 2021 | 4,828.84 USD | [10] |

| Unit | Unit value (standard error[a,b]) | Source |
|---|---|---|
| Cost of specialist care Diabetes consultation<br>code 910A | 169.01 USD (33.80) | [10] |
| Cost of fetal diagnostic examination<br>code 914Q | 135.21 USD (27.04) | [10] |
| Cost of outpatient consultation regarding pregnancy, childbirth, or postpartum period<br>Code 914O | 111.06 USD (22.21) | [10] |
| Cost of cesarean section with complications<br>code 370 | 9,169.96 USD (1,833.99) | [10] |
| Cost of cesarean section without complications<br>code 371 | 6,596.19 USD (1,319.24) | [10] |
| Cost of vaginal delivery with complications<br>code 372 | 4,249.38 USD (849.88) | [10] |
| Cost of vaginal delivery without complications<br>code 373 | 2,771.75 USD (554.35) | [10] |
| Cost of obstetric diagnostic measures<br>code 914P | 125.55 USD (25.11) | [10] |
| Cost of operation for anal sphincter injury | 1,515.39 USD (4.84) | [18 b] |
| Cost of fresh newborn<br>code 391 | 2,539.97 USD (507.99) | [10] |
| Prematurity cost increase | 45,782.21 USD (9,156.44) | See table A-2.9 |
| Cost of other delivery related procedures<br>code 814R | 371.82 USD (74.36) | [10] |
| Cost of newborn, birth weight 1500-2499g or other immaturity, with multiple problems<br>code 388A | 56,144.89 USD (11,228.98) | [10] |
| Cost of newborn, birth weight 2500g+ with multiple problems<br>code 389B | 22,009.84 USD (4,401.97) | [10] |
| Cost of newborn, birth weight >2500g with other significant problem<br>code 390 | 4,775.72 USD (955.14) | [10] |
| Cost of newborn, death within 0-2 days<br>code 385A | 6,572.05 USD (1,314.41) | [10] |

a: If no information on the underlying distribution was available, we assumed the standard error to be 20% of the mean value.
b: For costs in USD, $1 = 9.675 NOK purchasing power parities.
b: 1€ = 7.86 NOK, indexed from 2011 to 2021[22], then $1 = 9.675 NOK purchasing power parities.

Table A-2.8 Proportion of births with and without complications for vaginal delivery and cesarean section

| DRG code | % reported in 2018 | Proportion per type of birth |
|---|---|---|
| 373<br>Vaginal delivery without complications | 58% | 58/(58+23)=71.60% |
| 372<br>Vaginal delivery with complications | 23% | 23/(58/23)=28.40% |
| 371<br>Cesarean section without complications | 8% | 8/(8+8)=50.00% |

| | | |
|---|---|---|
| 370<br>Cesarean section with complications | 8% | 8/(8+8)=50.00% |
| 374, 373O, 375, 371O<br>Other | 3% | |

**Source:** For the cost parameter of vaginal delivery and cesarean section, we applied proportions weighs for deliveries with and without complications based on a report from the Norwegian Directorate of Health[14].

Table A-2.9 Calculation of weighted average reimbursement weight for pre-term birth in pre-eclampsia cases

| Newborn birth weight category | Number of newborns in 2020 | % of newborns in birth weight category | DRG code | DRG weight |
|---|---|---|---|---|
| <1000g | 211 | 8.8% | 386N newborn, birth weight below 1000g | 25.918 |
| 1000-1499g | 276 | 11.5% | 387N newborn, birth weight 1000-1499g | 17.362 |
| 1500-2499g | 1904 | 79.6% | 388A newborn, birth weight 1500-2499g or other immaturity, with multiple problems (assumed 50%) | 11.627 |
| | | | 388B newborn, birth weight 1500-2499g or other immaturity, without multiple problems (assumed 50%) | 2.729 |
| Total <2500g | 2391 | 100% | Weighted average from above | 10.007 |

**Source:** For the cost parameter of preeclampsia, we calculated the prematurity cost increase as the average of reimbursement weights for pre-term newborns[10]. Because prematurity is reflected by birth weight in the reimbursement code system, we weighted the DRG codes for pre-term newborns by the birth weight category proportions within birth weight below 2,500g reported in the Medical Birth Registry for 2020[16] multiplied with the DRG unit cost 2021 and minus the cost of fresh newborn

## Appendix 3 Short-term QALY values

Table A-3.1 Calculation of QALY decrements from short-term pregnancy outcomes

| Pregnancy outcome | QALY decrement | Calculation and elicitation of health state utility values | Source |
|---|---|---|---|
| Vaginal delivery | -0.0260 | Difference between utility value for vaginal delivery (0.7739, standard error 0.027) and perfect health (1.0000) over 42 days (standard error assumed 8.4) based on observed ceiling effect at 6 weeks<br>SF-36 questionnaire given to n=71 Dutch women 1 week after delivery<br>SF-36 scores mapped to EQ-5D utility values using a model based on UK TTO value set (model 3 including interaction) | [23,24] |
| Cesarean delivery | -0.0381 | Difference between utility value for cesarean section (0.6688, standard error 0.038) and perfect health (1.000) over 42 days (standard error assumed 8.4) based on observed ceiling effect at 6 weeks<br>SF-36 questionnaire given to n=70 Dutch women 1 week after delivery<br>SF-36 scores mapped to EQ-5D utility values using a model based on UK TTO value set | [23,24] |
| Preeclampsia | -0.0013 | Difference between utility value for preeclampsia (0.7574, standard error 0.024) and perfect health (1.0000) over 2 days (standard error assumed 0.4) based on clinical guidelines for induction of labor with preeclampsia<br>SF-12 questionnaire given to n=85 Austrian women who had preeclampsia in their pregnancy, proportion-weighed summary scores from mild and severe preeclampsia<br>SF-12 scores mapped to EQ-5D utility values using a model based on the UK TTO value set (2-variable model) | [15,25,26] |
| Induction of labor | +0.002 | Mean treatment difference in randomized clinical trial of labor induction near term versus expectant management<br>EQ-5D-5L questionnaire given to n=171 women in the UK in induction group of randomized clinical trial at week 39 and 1 month after delivery<br>QALYs estimated by the area-under-the-curve for individual patients using the cross-walk value set to the EQ-5D-3L UK TTO value set<br>Normal distribution of the treatment effect assumed for probabilistic analysis, standard error 0.0002 | [27] |
| Perineal trauma | -0.0398 | Difference between utility value with primary repair of anal sphincter injury (0.9193, standard error 0.025) and perfect health (1.0000) over 180 days (standard error assumed 36.0) based on recommended follow-up after 6-12 months in clinical guidelines<br>SF-36 questionnaire given to n=62 British women 3-12 months post-partum in randomized clinical trial group with primary repair of anal sphincter injury<br>SF-36 scores mapped to EQ-5D utility values using a model based on UK TTO value set (model 3 including interaction) | [15,24,28] |
| Shoulder dystocia | -0.0001 | An estimated 2.3-16% of shoulder dystocia cases lead to brachial plexus injury, midpoint chosen for QALY decrement<br>Difference between utility for brachial plexus injury that resolves within 2 months (0.99, standard error 0.017) and perfect health (1.0000) over 60 days (standard error assumed 12.0)<br>Utility value estimated by expert panel with 7 members | [15,29] |
| NICU admission – maternal perspective | -0.0030 | Difference between utility of 10-day intensive care unit hospitalization from the parental perspective (0.910, standard error 0.01) and perfect health (1.0000) over 12 days (standard error assumed 2.4) based on typical length of stay in neonatal units<br>Utility value elicited from n=403 parents through TTO method | [19,30] |

| | | | |
|---|---|---|---|
| NICU admission – neonatal perspective | -0.0030 | Difference between utility of pediatric intensive care for unspecified reasons (0.910, standard error 0.009) and perfect health (1.0000) over 12 days (standard error assumed 2.4) based on typical length of stay in neonatal units<br>Utility value from systematic review of childhood health utilities elicited through TTO method | [19,31] |
| Perinatal death – maternal perspective | -0.10990 | Difference between utility of perinatal death from the maternal perspective (0.777, standard error 0.014) and perfect health (1.0000) over 180 days (standard error assumed 36.0)<br>Utility value based on index score from EQ-5D-5L questionnaire given to n=112 British women at week 15 of next pregnancy after perinatal death in previous pregnancy<br>Mean and standard error calculated from reported median and interquartile range based on the method by Wan et al. (2014) | [32,33] |

Table A-3.2 Structured literature review for short-term QALY values

| Parameter and date of search | Results from search in databases | Inclusion criteria | Results after scanning title and abstract -duplicates +cross-references | Results after scanning full text |
|---|---|---|---|---|
| 1. Utility value for preeclampsia 31.03.2022 | A:97<br>B:92 | Information about utility value or disutility associated with the chosen perinatal complication<br>Information included in cost-utility analysis which refers to another study, or a different type of study<br>Information about the population and the elicitation method<br>Information about research design, data source and potential bias<br>Excluding cost-effectiveness analyses without QALYs, study protocols and studies that are based on questionnaires for which no standard utility value set, or mapping is available<br>Excluding long-term studies measuring quality of life more than 3 months post-partum and studies from settings deemed very different from Norway (e.g., low-income rural settings) | A:3<br>B:1 | 1 |
| 2. Utility value for cesarean section 31.03.2022 | A: 17<br>B:117 | | A:4<br>B:4<br>-1<br>+6 | 5 |
| 3. Utility value for gestational hypertension 31.03.2022 | A:110<br>B:79 | | A:4<br>B:2<br>-3<br>+2 | 0 |
| 4. Utility value for induction of labor 04.04.2022 | A:44<br>B:84 | | A:7<br>B:12<br>-6 | 2 |
| 5. Utility value for perineal trauma 04.04.2022 | A:84<br>B:169 | | A:4<br>B:5<br>-1<br>+1 | 2 |
| 6. Utility value for shoulder dystocia 05.04.2022 | A:12<br>B:13 | | A:3<br>B:7<br>-3<br>+2 | 2 |
| 7. Utility value for neonatal intensive care unit admission 05.04.2022 | A:159<br>B:155 | | A:7<br>B:7<br>-7<br>+3 | 5 |
| 8. Utility value for neonatal respiratory distress syndrome 06.04.2022 | A:22<br>B:12 | | A:1<br>B:3<br>+3 | 3 |
| 9. Utility value for neonatal hypoglycemia 06.04.2022 | A:5<br>B:9 | | A:1<br>B:2<br>-1 | 0 |
| 10. Utility value for neonatal hyperbilirubinemia 06.04.2022 | A:10<br>B:11 | | A:2<br>B:1<br>-1<br>+1 | 1 |
| 11. Utility value for neonatal death – maternal perspective 07.04.2022 +08.04.2022 | A:69<br>B:101 | | A:13<br>B:17<br>-8<br>+3 | 6 |

1a. ("pre eclampsia"[MeSH Terms] OR "pre-eclampsia"[Title/Abstract]) AND ("quality of life"[MeSH Terms] OR “utility value*”[Title/Abstract] OR "quality of life"[Title/Abstract] OR

“QALY*”[Title/Abstract] OR “health state valuation”[Title/Abstract] OR “well-being”[Title/Abstract]) AND ("english"[Language] OR "german"[Language]) AND (y_10[Filter])

1b. (AB=(pre-eclampsia)) AND (AB=(utility value) OR AB=(quality of life) OR AB=(QALY*) OR AB=(health state valuation) OR AB=(QALY) OR AB=(well-being)) AND (LA=(English) OR LA=(German))

Filter: Publication date last 10 years

2a. ("cesarean section"[MeSH Terms] OR "cesarean section"[Title/Abstract]) AND (“utility value*”[Title/Abstract] OR “QALY*”[Title/Abstract] OR “health state valuation”[Title/Abstract]) AND ("english"[Language] OR "german"[Language]) AND (y_10[Filter])

2b. (AB=(cesarean section)) AND (AB=(utility value) OR AB=(quality of life) OR AB=(QALY*) OR AB=(health state valuation) OR AB=(QALY)) AND (LA=(English) OR LA=(German))

Filter: Publication date last 10 years

3a. ("hypertension, pregnancy-induced"[MeSH Terms] OR "gestational hypertension"[Title/Abstract] OR “pregnancy-induced hypertension”[Title/Abstract]) AND ("quality of life"[MeSH Terms] OR “utility value*”[Title/Abstract] OR "quality of life"[Title/Abstract] OR “QALY*”[Title/Abstract] OR “health state valuation”[Title/Abstract] OR “well-being”[Title/Abstract]) AND ("english"[Language] OR "german"[Language]) AND (y_10[Filter])

3b. (AB=(gestational hypertension) OR AB=(pregnancy-induced hypertension)) AND (AB=(utility value) OR AB=(quality of life) OR AB=(QALY*) OR AB=(health state valuation) OR AB=(QALY)) AND (LA=(English) OR LA=(German))

Filter: Publication date last 10 years

4a. ("labor, induced"[MeSH Terms] OR "induced labor"[Title/Abstract] OR “induction of labor”[Title/Abstract] OR “induced birth”[Title/Abstract] OR “induction of birth”[Title/Abstract]) AND ("quality of life"[MeSH Terms] OR “utility value*”[Title/Abstract] OR "quality of life"[Title/Abstract]OR “QALY*”[Title/Abstract] OR “health state valuation”[Title/Abstract] OR “well-being”[Title/Abstract]) AND ("english"[Language] OR "german"[Language]) AND (y_10[Filter])

4b. (AB=(induced labor) OR AB=(induction of labor) OR AB=(induction of birth)) AND (AB=(utility value) OR AB=(quality of life) OR AB=(QALY*) OR AB=(health state valuation) OR AB=(QALY)) AND (LA=(English) OR LA=(German))

Filter: Publication date last 10 years

5a. ("perineal trauma"[Title/Abstract] OR “perineal tear”[Title/Abstract] OR “perineal rupture”[Title/Abstract] OR “birth rupture”[Title/Abstract] OR “maternal birth injury”[Title/Abstract] OR “anal sphincter injury”[Title/Abstract]) AND ("quality of life"[MeSH Terms] OR “utility value*”[Title/Abstract] OR "quality of life"[Title/Abstract] OR “QALY*”[Title/Abstract] OR “health state valuation”[Title/Abstract] OR “well-being”[Title/Abstract]) AND ("english"[Language] OR "german"[Language]) AND (y_10[Filter])

5b. (AB=(perineal trauma) OR AB=(perineal tear) OR AB=(perineal rupture) OR AB=(birth rupture) OR AB=(maternal birth injury) OR AB=(anal sphincter injury)) AND (AB=(utility value) OR AB=(quality of life) OR AB=(QALY*) OR AB=(health state valuation) OR AB=(QALY)) AND (LA=(English) OR LA=(German))

Filter: Publication date last 10 years

6a. ("shoulder dystocia"[MesH Terms] OR “shoulder dystocia”[Title/Abstract]) AND ("quality of life"[MeSH Terms] OR “utility value*”[Title/Abstract] OR "quality of life"[Title/Abstract] OR “QALY*”[Title/Abstract] OR “health state valuation”[Title/Abstract] OR “well-being”[Title/Abstract]) AND ("english"[Language] OR "german"[Language]) AND (y_10[Filter])

6b. (AB=(shoulder dystocia)) AND (AB=(utility value) OR AB=(quality of life) OR AB=(QALY*) OR AB=(health state valuation) OR AB=(QALY)) AND (LA=(English) OR LA=(German))

Filter: Publication date last 10 years

7a. ("intensive care units, neonatal"[MeSH Terms] OR "NICU admission*"[Title/Abstract] OR “neonatal intensive care”[Title/Abstract]) AND ("quality of life"[MeSH Terms] OR “utility value*”[Title/Abstract] OR "quality of life"[Title/Abstract] OR “QALY*”[Title/Abstract] OR “health state valuation”[Title/Abstract] OR “well-being”[Title/Abstract]) AND ("english"[Language] OR "german"[Language]) AND (y_10[Filter])

7b. (AB=(NICU admission) OR AB=(neonatal intensive care)) AND (AB=(“utility value”) OR AB=(“quality of life”) OR AB=(QALY*) OR AB=(“health state valuation”) OR AB=(“QALY”)) AND (LA=(English) OR LA=(German))

Filter: Publication date last 10 years

8a. ("respiratory distress syndrome, newborn"[MeSH Terms] OR "neonatal respiratory distress syndrome"[Title/Abstract] OR “newborn respiratory distress syndrome”[Title/Abstract]) AND ("quality of life"[MeSH Terms] OR “utility value*”[Title/Abstract] OR "quality of life"[Title/Abstract] OR “QALY*”[Title/Abstract] OR “health state valuation”[Title/Abstract] OR “well-being”[Title/Abstract]) AND ("english"[Language] OR "german"[Language]) AND (y_10[Filter])

8b. (AB=(newborn respiratory distress syndrome) OR AB=(neonatal respiratory distress syndrome)) AND (AB=("utility value") OR AB=("quality of life") OR AB=(QALY*) OR AB=("health state valuation") OR AB=("QALY")) AND (LA=(English) OR LA=(German))

Filter: Publication date last 10 years

9a. ("neonatal hypoglycemia"[Title/Abstract] OR "newborn hypoglycemia"[Title/Abstract]) AND ("quality of life"[MeSH Terms] OR "utility value*"[Title/Abstract] OR "quality of life"[Title/Abstract] OR "QALY*"[Title/Abstract] OR "health state valuation"[Title/Abstract] OR "well-being"[Title/Abstract]) AND ("english"[Language] OR "german"[Language]) AND (y_10[Filter])

9b. (AB=(newborn hypoglycemia) OR AB=(neonatal hypoglycemia)) AND (AB=("utility value") OR AB=("quality of life") OR AB=(QALY*) OR AB=("health state valuation") OR AB=("QALY")) AND (LA=(English) OR LA=(German))

Filter: Publication date last 10 years

10a. ("hyperbilirubinemia, neonatal"[MeSH Terms] OR "neonatal hyperbilirubinemia"[Title/Abstract] OR "neonatal jaundice"[Title/Abstract] OR "newborn jaundice"[Title/Abstract] OR "newborn hyperbilirubinemia"[Title/Abstract) AND ("quality of life"[MeSH Terms] OR "utility value*"[Title/Abstract] OR "quality of life"[Title/Abstract] OR "QALY*"[Title/Abstract] OR "health state valuation"[Title/Abstract] OR "well-being"[Title/Abstract]) AND ("english"[Language] OR "german"[Language]) AND (y_10[Filter])

10b. (AB=(neonatal hyperbilirubinemia) OR AB=(neonatal jaundice) OR AB=(newborn hyperbilirubinemia) OR AB=(newborn jaundice)) AND (AB=("utility value") OR AB=("quality of life") OR AB=(QALY*) OR AB=("health state valuation") OR AB=("QALY")) AND (LA=(English) OR LA=(German))

Filter: Publication date last 10 years

11a. ("infant mortality"[MeSH Terms] OR "neonatal mortality"[Title/Abstract] OR "newborn mortality"[Title/Abstract] OR "stillbirth"[Title/Abstract]) AND ("quality of life"[MeSH Terms] OR "utility value*"[Title/Abstract] OR "quality of life"[Title/Abstract] OR "QALY*"[Title/Abstract] OR "health state valuation"[Title/Abstract]) AND ("english"[Language] OR "german"[Language]) AND (y_10[Filter])

11b. (AB=("infant mortality") OR AB=("neonatal mortality") OR AB=("newborn mortality") OR AB=(stillbirth)) AND (AB=("utility value") OR AB=("quality of life") OR AB=(QALY*) OR AB=("health state valuation") OR AB=("QALY")) AND (LA=(English) OR LA=(German))

Filter: Publication date last 10 years

## Appendix 4 Calculation of long-term costs and QALY loss

Table A-4.1 Parameters for the calculation of long-term costs and QALY loss

| Parameter | Value (standard error[a,b]) | Source |
|---|---|---|
| Annual direct medical costs of diabetes | 4,612.88 USD (922.58) | 34 |
| Annual costs of drugs and diabetes specific medical consumables | 2,759.85 USD (551.97) | 34 |
| Annual patient time costs for glucose self-measuring assuming 5 measures per day with 5 mins duration and the usual time cost per hour of 26 USD | 3,999.19 USD (799.84) | 7 |
| Utility decrement from type 2 diabetes Catalogue value elicited using EQ-5D questionnaires from 79,522 individuals from the US valued with the UK population value set | -0.0621 (0.0038) | 35 |

a: If no information on the underlying distribution was available, we assumed the standard error to be 20% of the mean value.
b: 1€ = 7.86 NOK, indexed from 2011 to 2021[22], then $1 = 9.675 NOK purchasing power parities.

For the lifetime calculation of the costs and QALY loss from later maternal diabetes (Table A-4.2), we assumed that maternal age at childbirth was 30. The remaining life expectancy at 30 years in Norway is 55 years[36], and we assumed that eight years of life would be lost due to diabetes[37]. Since the systematic review by Brown and colleagues[38] reported the relative risk of development of type 2 diabetes at a maximum of 10 years after giving birth, we assumed half of this timeframe, five years, to be the average number of years before diabetes onset. For the utility values without diabetes, we applied the utility values for the general population from the Norwegian Institute of Public Health[39]. For the utility values with diabetes, we applied the catalogue utility decrement from Table A-4.1 to the utility values without diabetes, starting after five years. We applied a discount rate of 4% for both costs and QALYs[39]. We applied the total diabetes-related costs and the QALY increment to the outcome of maternal diabetes type 2 in the model.

Table A-4.2 Calculation of lifetime costs and QALY loss from later maternal diabetes type 2

| Timepoint | Age | Diabetes-related costs per year in USD[a] | Discounted costs per year in USD[a] | Utility healthy | Discounted QALYs healthy | Utility with diabetes | Discounted QALYs with diabetes |
|---|---|---|---|---|---|---|---|
| 0 | 30 | 0 | 0 | 0.906 | 0.9060 | 0.906 | 0.906 |
| 1 | 31 | 0 | 0 | 0.87 | 0.8365 | 0.87 | 0.8365 |
| 2 | 32 | 0 | 0 | 0.87 | 0.8044 | 0.87 | 0.8044 |
| 3 | 33 | 0 | 0 | 0.87 | 0.7734 | 0.87 | 0.7734 |
| 4 | 34 | 0 | 0 | 0.87 | 0.7437 | 0.87 | 0.7437 |
| 5 | 35 | 11,371.92 | 9,346.89 | 0.87 | 0.7151 | 0.8079 | 0.6640 |
| 6 | 36 | 11,371.92 | 8,987.39 | 0.87 | 0.6876 | 0.8079 | 0.6385 |
| | | | … | | | | |
| 44 | 74 | 11,371.92 | 2,024.73 | 0.808 | 0.1439 | 0.7459 | 0.1328 |
| 45 | 75 | 11,371.92 | 1,946.85 | 0.808 | 0.1383 | 0.7459 | 0.1277 |
| 46 | 76 | | | 0.808 | 0.1330 | | |
| 47 | 77 | | | 0.808 | 0.1279 | | |
| 48 | 78 | | | 0.808 | 0.1230 | | |
| 49 | 79 | | | 0.808 | 0.1182 | | |
| 50 | 80 | | | 0.808 | 0.1137 | | |
| 51 | 81 | | | 0.73 | 0.0988 | | |
| 52 | 82 | | | 0.73 | 0.0950 | | |
| 53 | 83 | | | 0.73 | 0.0913 | | |
| | | | **194,347.74** | | **19.28** | | **17.32** |
| | | | | | | **Increment -1.96** | |

a: $1 = 9.675 NOK purchasing power parities

For the long-term loss in QALYs related to perinatal death (Table A-4.3), we assumed that a newborn would on average live for 83 years, based on the life expectancy at birth for both genders in Norway.[36] We then applied the utility values for the general population from the Norwegian Institute of Public Health[39] and the discount rate of 4%[39] to derive the total discounted QALYs for a healthy newborn over a lifetime horizon. The negative value of this sum represents the QALY loss from perinatal death in the model.

Table A-4.3 Calculation of lifetime QALY loss from perinatal death

| Timepoint | Age | Utility healthy | Discounted QALYs healthy |
|---|---|---|---|
| 0 | 0 | 0.926 | 0.9260 |
| 1 | 1 | 0.926 | 0.8904 |
| 2 | 2 | 0.926 | 0.8561 |
| 3 | 3 | 0.926 | 0.8232 |
| 4 | 4 | 0.926 | 0.7915 |
| | | … | |
| 80 | 80 | 0.808 | 0.0351 |
| 81 | 81 | 0.73 | 0.0305 |
| 82 | 82 | 0.73 | 0.0293 |
| 83 | 83 | 0.73 | 0.0282 |
| | | | **22.51** |

## Appendix 5 Scenario analysis

Table A-5.1 Scenarios in scenario analysis

| Scenario | Description of model changes for the scenario |
|---|---|
| 1) Higher GDM prevalence | Prevalence parameter values increased from 10.3%/11.6%/27.0% to 20.3%/21.6%/37.0%, fixed for probabilistic analysis |
| 2) Lower screening adherence | Screening adherence decreased from 100% to 73.0% in all screening strategies, fixed for probabilistic analysis<br>New value resulted from calibrating screening adherence with current screening to 5.9% of women diagnosed with GDM in the Medical Birth Registry in 2020<br>5.9% split into 4.66% and 1.24% of women in treatment category C and D assuming the same ratio of lifestyle/pharmacological treatment<br>Excel goal seek function resulted in 73.0% screening adherence |
| 3) Lower percentage requiring further treatment with universal screening | Parameter value for the proportion of women referred to specialist services for treatment review decreased from 35% to 20% for universal screening only, for current, high-risk and no-screening set to 35% parameter fixed for probabilistic analysis |
| 4) Perfect screening test characteristics | Parameters for sensitivity and specificity increased from 95% to 100%, fixed for probabilistic analysis |
| 5) Unfavorable treatment effect | Relative risk of adverse pregnancy outcomes with treated versus untreated GDM set to the upper bound of the 95% confidence interval, fixed for probabilistic analysis |
| 6) More favorable treatment effect | Relative risk of adverse pregnancy outcomes with treated versus untreated GDM set to the lower bound of the 95% confidence interval, fixed for probabilistic analysis |
| 7) Lower risk of adverse pregnancy outcomes associated with GDM | Relative risk of adverse pregnancy outcomes with GDM versus normal glucose tolerance set to the lower bound of the 95% confidence interval, fixed for probabilistic analysis |
| 8) Higher risk of adverse pregnancy outcomes associated with GDM | Relative risk of adverse pregnancy outcomes with GDM versus normal glucose tolerance set to the upper bound of the 95% confidence interval, fixed for probabilistic analysis |
| 9) Alternative set of probabilities with untreated GDM | Instead of relative risk rates, using the reported probability of adverse pregnancy outcomes with treated and untreated GDM from a systematic review of GDM treatment trials[40] |
| 10) Alternative set of QALY decrements | Parameters for QALY loss set to the ones reported by Farrar and colleagues[3] and fixed for probabilistic analysis:<br>- 0.00456 for preeclampsia<br>- 0.0017 for cesarean section<br>- 2.05 for shoulder dystocia<br>- 0.053 for perineal trauma<br>QALY loss from all other short-term adverse perinatal outcomes set to zero<br>QALY loss from perinatal death as before |
| 11) Healthcare perspective | Parameter values for patient time cost per hour and patient travel cost set from USD 26 and USD 24 to zero, fixed for probabilistic analysis |
| 12) Including large for gestational age and dependent probabilities | Large for gestational age newborn included as outcome, assuming dependent probabilities for cesarean section, shoulder dystocia, perineal trauma, and neonatal hypoglycemia<br>Details see Table A-5.2 |
| 13) Excluding perinatal mortality | Probability of perinatal death set to zero, independent of GDM treatment status |

Under scenario 12, we included large for gestational age (LGA) as additional disease outcome with dependent probabilities for the risk of cesarean section, shoulder dystocia, perineal trauma, and neonatal hypoglycemia (Table A-5.2). Due to lack of reported treatment effect rates stratified by the outcome GDM, we assumed that GDM treatment has a direct treatment effect on the outcome LGA, but not directly on cesarean section, shoulder dystocia, perineal trauma, and neonatal hypoglycemia in combination with LGA. The probabilities of complications without LGA were defined as in the base case.

Table A-5.2 Parameter details for scenario 12 including large for gestational age

| Parameter | Value | Source |
|---|---|---|
| **Values reported as x/N \| probability or relative risk rate [95 % confidence interval] or probability** | | |
| Probability of LGA with normal glucose tolerance | 1515/9935 \| 15.2% | [41-43] |
| Relative risk rate of LGA associated with GDM versus normal glucose tolerance | 1.69 [1.42-2.01] | [40] |
| Probability of LGA with untreated GDM | 25.8% | Calculated from above |
| Relative risk rate of LGA associated with GDM treatment versus no treatment | 0.56 [0.47-0.66] | [40] |
| Probability of LGA with treated GDM | 14.4% | Calculated from above |
| Risk of cesarean section with LGA and normal glucose tolerance | 16,940/83,493 \| 20.3% | [44] |
| Risk of cesarean section with LGA and GDM (treated and untreated) | 555/1,547 \| 35.9% | [44] |
| Risk of shoulder dystocia with LGA and normal glucose tolerance | 190/3,167 \| 6.0% | [45] |
| Risk of shoulder dystocia with LGA and GDM (treated and untreated) | 16/152 \| 10.5% | [45] |
| Risk of perineal trauma with LGA and normal glucose tolerance | 2,107/83,493 \| 2.5% | [44] |
| Risk of perineal trauma with LGA and GDM (treated and untreated) | 57/1,547 \| 3,7% | [44] |
| Risk of neonatal hypoglycemia with LGA and normal glucose tolerance | 1,422/83,493 \| 1.7% | [44] |
| Risk of neonatal hypoglycemia with LGA and GDM (treated and untreated) | 99/1,547 \| 6.4% | [44] |

Table A-5.3 Probabilistic model results in scenario analysis

| | Total costs | Total QALY (loss) | ∆ Total costs | ∆ Total QALY (gain) | ICER |
|---|---|---|---|---|---|
| **1) Higher GDM prevalence** | | | | | |
| No-screening | $ 641,624,079 | - 7,361 | | | |
| High-risk screening | $ 643,512,058 | - 6,824 | $ 1,887,979 | 537 | $ 3,519 |
| Current screening | $ 649,779,571 | - 6,169 | $ 6,267,513 | 655 | Extended dominance |
| Universal screening | $ 652,891,002 | - 5,829 | $ 3,111,431 | 340 | **$ 9,422** |
| **2) Lower screening adherence** | | | | | |
| No-screening | $ 637,021,668 | - 7,027 | | | |
| High-risk screening | $ 638,518,447 | - 6,741 | $ 1,496,779 | 286 | $ 5,230 |
| Current screening | $ 643,435,832 | - 6,558 | $ 4,917,385 | 183 | Extended dominance |
| Universal screening | $ 645,875,439 | - 6,457 | $ 2,439,606 | 101 | **$ 25,965** |
| **3) Lower percentage requiring further treatment with universal screening** | | | | | |
| No-screening | $636,523,443 | - 7,010 | | | |
| High-risk screening | $638,586,648 | - 6,619 | $2,063,205 | 391 | $ 5,283 |
| Current screening | $645,326,310 | - 6,370 | $6,739,662 | 249 | Extended dominance |
| Universal screening | $648,520,930 | - 6,231 | $3,194,620 | 138 | **$ 25,620** |
| **4) Perfect screening test characteristics** | | | | | |
| No-screening | $ 402,001 | - 7,037 | | | |
| High-risk screening | $ 38,099,808 | - 6,625 | $ 1,697,807 | 411 | $ 4,127 |
| Current screening | $ 643,596,545 | - 6,362 | $ 5,496,736 | 263 | Extended dominance |
| Universal screening | $ 646,328,957 | - 6,218 | $ 2,732,413 | 144 | **$ 20,180** |
| **5) Unfavorable treatment effect** | | | | | |
| No-screening | $ 637,184,812 | - 7,043 | | | **Cost-effective** |
| High-risk screening | $ 642,530,907 | - 7,343 | $ 5,346,094 | - 301 | $ -17,777 |
| Current screening | $ 651,376,897 | - 7,536 | $ 8,845,991 | - 193 | Extended dominance |
| Universal screening | $ 655,884,322 | - 7,642 | $ 4,507,425 | - 106 | $ -44,672 |
| **6) More favorable treatment effect** | | | | | |
| No-screening | $ 636,537,215 | - 7,035 | | | |

| | | | | | |
|---|---|---|---|---|---|
| High-risk screening | $ 636,087,202 | - 6,608 | $ -450,013 | 427 | $ -1,053 |
| Current screening | $ 641,222,264 | - 6,335 | $ 5,135,062 | 273 | Extended dominance |
| Universal screening | $ 643,685,728 | - 6,184 | $ 2,463,464 | 151 | **$ 17,932** |
| **7) Lower risk of adverse pregnancy outcomes associated with GDM** | | | | | |
| No-screening | $ 632,174,769 | - 6,623 | | | |
| High-risk screening | $ 634,715,724 | - 6,404 | $ 2,540,955 | 219 | **$ 11,621** |
| Current screening | $ 641,762,764 | - 6,264 | $ 7,047,040 | 140 | Extended dominance |
| Universal screening | $ 645,279,485 | - 6,186 | $ 3,516,721 | 77 | $ 48,492 |
| **8) Higher risk of adverse pregnancy outcomes associated with GDM** | | | | | |
| No-screening | $ 642,214,175 | - 7,725 | | | |
| High-risk screening | $ 643,700,512 | - 7,034 | $ 1,486,337 | 691 | $ 2,152 |
| Current screening | $ 650,063,642 | - 6,594 | $ 6,363,130 | 440 | Extended dominance |
| Universal screening | $ 653,199,652 | - 6,349 | $ 3,136,010 | 245 | **$ 13,865** |
| **9) Alternative set of probabilities with untreated GDM** | | | | | |
| No-screening | $ 646,474,696 | - 6,522 | | | |
| High-risk screening | $ 648,636,978 | - 6,395 | $ 2,162,282 | 127 | **$ 17,089** |
| Current screening | $ 655,443,071 | - 6,314 | $ 6,806,093 | 81 | Extended dominance |
| Universal screening | $ 658,826,190 | - 6,269 | $ 3,383,120 | 45 | $ 81,008 |
| **10) Alternative set of QALY decrements** | | | | | |
| No-screening | $ 636,101,522 | - 6,994 | | | |
| High-risk screening | $ 638,159,481 | - 6,543 | $ 2,057,959 | 451 | $ 4,566 |
| Current screening | $ 644,900,777 | - 6,254 | $ 6,741,296 | 289 | Extended dominance |
| Universal screening | $ 648,250,984 | - 6,096 | $ 3,350,207 | 158 | **$ 22,579** |
| **11) Healthcare perspective** | | | | | |
| No-screening | $ 503,016,885 | - 7,020 | | | |
| High-risk screening | $ 502,743,447 | - 6,629 | $ -273,438 | 392 | $ -698 |
| Current screening | $ 505,008,357 | - 6,379 | $ 2,264,910 | 250 | Extended dominance |
| Universal screening | $ 506,087,582 | - 6,240 | $ 1,079,226 | 139 | **$ 8,598** |
| **12) Including large for gestational age and dependent probabilities** | | | | | |
| No-screening | $ 641,274,010 | - 7,049 | | | |

| | | | | | |
|---|---|---|---|---|---|
| High-risk screening | $ 643,024,398 | - 6,655 | $ 1,750,388 | 394 | $ 4,444 |
| Current screening | $ 649,567,218 | - 6,403 | $ 6,542,821 | 252 | Extended dominance |
| Universal screening | $ 652,803,866 | - 6,263 | $ 3,236,647 | 139 | **$ 24,971** |
| **13) Excluding perinatal mortality** | | | | | |
| No-screening | $ 637,051,503 | - 1,435 | | | **Cost-effective** |
| High-risk screening | $ 639,123,889 | - 1,434 | $ 2,072,385 | 1 | $ 2,177,166 |
| Current screening | $ 645,884,108 | - 1,433 | $ 6,760,219 | 1 | Extended dominance |
| Universal screening | $ 649,240,987 | - 1,433 | $ 3,356,878 | 0 | $ 10,712,961 |

## Appendix 6 Model validation

Table A-6.1 Validation of probability parameters for adverse pregnancy outcomes with treated GDM

| | Probability of outcome with treated GDM (see also Table 2) | Proportion of outcome among women with diagnosed GDM in the Medical Birth Registry 2018-2020[6] |
|---|---|---|
| **Values reported as proportion [95% confidence interval]** | | |
| Cesarean section | 17.0% | 22.4% |
| Preeclampsia | 3.4% | 4.0% |
| Induction of labor | 29.0% | 53.3% |
| Perineal trauma (sphincter rupture grade 3-4)[a] | 1.7% | 1.2% |
| Admission to NICU or neonatology | 8.3% | 21.9% |
| Data on the remaining outcomes included in the model was not available in the Medical Birth Registry. | | |

Table A-6.2 Validation of model results in the current screening strategy with Norwegian registry data

| | Modelled proportion of outcome with current screening | Proportion of outcome among all births in the Medical Birth Registry 2018-2020 |
|---|---|---|
| **Values reported as proportion [95% confidence interval]** | | |
| Cesarean section | 15.17% [15.16-15.19] | 15.27% |
| Preeclampsia | 3.07% [3.07-3.08] | 2.45% |
| Induction of labor | 22.42% [22.41-22.44] | 25.07% |
| Perineal trauma (sphincter rupture grade 3-4) | 1.38% [1.38-1.38] | 1.34% |
| Admission to NICU or neonatology | 9.65% [9.64-9.66] | 9.80% |
| Data on the remaining outcomes included in the model was not available in the Medical Birth Registry. | | |

## Appendix 7 CHEERS reporting checklist[46]

| Topic | No. | Item | Location where item is reported |
|---|---|---|---|
| **Title** | | | |
| | 1 | Identify the study as an economic evaluation and specify the interventions being compared. | Title: "cost-effectiveness modeling of gestational diabetes screening" |
| **Abstract** | | | |
| | 2 | Provide a structured summary that highlights context, key methods, results, and alternative analyses. | Abstract |
| **Introduction** | | | |
| **Background and objectives** | 3 | Give the context for the study, the study question, and its practical relevance for decision making in policy or practice. | Introduction |
| **Methods** | | | |
| **Health economic analysis plan** | 4 | Indicate whether a health economic analysis plan was developed and where available. | Not applicable, study is model-based not trial-based |
| **Study population** | 5 | Describe characteristics of the study population (such as age range, demographics, socioeconomic, or clinical characteristics). | Section Methods, Analytic overview |
| **Setting and location** | 6 | Provide relevant contextual information that may influence findings. | Section Methods, Analytic overview |
| **Comparators** | 7 | Describe the interventions or strategies being compared and why chosen. | Section Methods, Screening strategies |
| **Perspective** | 8 | State the perspective(s) adopted by the study and why chosen. | Section Methods, Analytic overview |

| Topic | No. | Item | Location where item is reported |
|---|---|---|---|
| **Time horizon** | 9 | State the time horizon for the study and why appropriate. | Section Methods, Analytic overview |
| **Discount rate** | 10 | Report the discount rate(s) and reason chosen. | Section Methods, Analytic overview |
| **Selection of outcomes** | 11 | Describe what outcomes were used as the measure(s) of benefit(s) and harm(s). | Section Methods, Analytic overview |
| **Measurement of outcomes** | 12 | Describe how outcomes used to capture benefit(s) and harm(s) were measured. | Section Methods, Analytic overview and Model structure and disease outcomes |
| **Valuation of outcomes** | 13 | Describe the population and methods used to measure and value outcomes. | Section Methods, Cost and health-related quality of life assumptions |
| **Measurement and valuation of resources and costs** | 14 | Describe how costs were valued. | Section Methods, Cost and health-related quality of life assumptions |
| **Currency, price date, and conversion** | 15 | Report the dates of the estimated resource quantities and unit costs, plus the currency and year of conversion. | Section Methods, Analytic overview and Cost and health-related quality of life assumptions |
| **Rationale and description of model** | 16 | If modelling is used, describe in detail and why used. Report if the model is publicly available and where it can be accessed. | Section Methods, Model structure and disease outcomes |
| **Analytics and assumptions** | 17 | Describe any methods for analysing or statistically transforming data, any extrapolation methods, and approaches for validating any model used. | Methods, Analytic overview and Model structure and disease outcomes |
| **Characterising heterogeneity** | 18 | Describe any methods used for estimating how the results of the study vary for subgroups. | Not applicable |

| Topic | No. | Item | Location where item is reported |
|---|---|---|---|
| **Characterising distributional effects** | 19 | Describe how impacts are distributed across different individuals or adjustments made to reflect priority populations. | Not applicable |
| **Characterising uncertainty** | 20 | Describe methods to characterise any sources of uncertainty in the analysis. | Section Methods, Analytic overview |
| **Approach to engagement with patients and others affected by the study** | 21 | Describe any approaches to engage patients or service recipients, the general public, communities, or stakeholders (such as clinicians or payers) in the design of the study. | Not applicable |
| **Results** | | | |
| **Study parameters** | 22 | Report all analytic inputs (such as values, ranges, references) including uncertainty or distributional assumptions. | Section Methods, Analytic overview, Epidemiological and effect data, Cost and health-related quality of life assumptions, Table 1, Table 2, Appendix 2 Parameter details, Appendix 3 Short-term QALY values, Appendix 4 Calculation of long-term costs and QALY loss |
| **Summary of main results** | 23 | Report the mean values for the main categories of costs and outcomes of interest and summarise them in the most appropriate overall measure. | Section Results, Primary analysis, Secondary analysis, Table 3, Figure 2 |
| **Effect of uncertainty** | 24 | Describe how uncertainty about analytic judgments, inputs, or projections affect findings. Report the effect of choice of discount rate and time horizon, if applicable. | Section Results, Value of information analysis and Scenario analysis, Figure 3 |

| Topic | No. | Item | Location where item is reported |
|---|---|---|---|
| **Effect of engagement with patients and others affected by the study** | 25 | Report on any difference patient/service recipient, general public, community, or stakeholder involvement made to the approach or findings of the study | Not applicable |
| **Discussion** | | | |
| **Study findings, limitations, generalisability, and current knowledge** | 26 | Report key findings, limitations, ethical or equity considerations not captured, and how these could affect patients, policy, or practice. | Section Discussion |
| **Other relevant information** | | | |
| **Source of funding** | 27 | Describe how the study was funded and any role of the funder in the identification, design, conduct, and reporting of the analysis | Article footnotes |
| **Conflicts of interest** | 28 | Report authors conflicts of interest according to journal or International Committee of Medical Journal Editors requirements. | Article footnotes |

## References


1. Rai AS, Sletner L, Jenum AK, et al. Identifying women with gestational diabetes based on maternal characteristics: an analysis of four Norwegian prospective studies. *BMC Pregnancy Childbirth*. 2021;21(1):615. doi:10.1186/s12884-021-04086-9
2. Coop C, Edlin R, Brown J, Farquhar C. Cost-effectiveness of the New Zealand diabetes in pregnancy guideline screening recommendations. *BMJ Open*. 2015;5(6):e006996. doi:10.1136/bmjopen-2014-006996
3. Farrar D, Simmonds M, Griffin S, et al. The identification and treatment of women with hyperglycaemia in pregnancy: an analysis of individual participant data, systematic reviews, meta-analyses and an economic evaluation. *Health Technol Assess*. 2016;20(86):1-348. doi:10.3310/hta20860
4. Helsedirektoratet. Svangerskapsdiabetes Nasjonal faglig retningslinje. Updated January 10, 2022. Accessed January 18, 2022. https://www.helsedirektoratet.no/retningslinjer/svangerskapsdiabetes#referere
5. Oslo Economics. *Screening for svangerskapsdiabetes: En helseøkonomisk analyse av alternative målgrupper for screening*. 2021. Accessed January 18, 2022. https://osloeconomics.no/en/publication/screening-for-svangerskapsdiabetes/
6. Data from: Medical Birth Registry [aggregated data]. 2022. Deposited February 14, 2022 and March 25, 2022.
7. Statens legemiddelverk. Enhetskostnadsdatabase. Updated January 24, 2022. Accessed March 16, 2022, 2022. https://legemiddelverket.no/offentlig-finansiering/dokumentasjon-for-metodevurdering/enhetskostnadsdatabase
8. Legeforeningen. Takster for allmennpraktiserende lege i fastlegeordningen. Accessed March 16, 2022. https://normaltariffen.legeforeningen.no/book/Fastlegetariffen-2021/m-618
9. Senter for klinisk dokumentasjon og evaluering. *Helseatlas for fødselshjelp 2015-2017*. 2019. Accessed March 16, 2022. https://helseatlas.no/sites/default/files/helseatlas-fodselshjelp.pdf
10. Helsedirektoratet. *Regelverk for innsatsstyrt finansiering 2021 (ISF-regelverket)*. 2020. Accessed March 19, 2022. https://www.helsedirektoratet.no/tema/finansiering/innsatsstyrt-finansiering-og-drg-systemet/innsatsstyrt-finansiering-isf/ISF-regelverket%202021.pdf/_/attachment/inline/3e084cec-bce9-4a44-89f0-f8d179b4e478:5229e063e72719e76f57316d73d45ee57fb60a96/ISF-regelverket%202021.pdf
11. Helfo. Produkt- og prislister. Accessed March 19, 2022. https://www.helfo.no/produkt-og-prislister/produkt-og-prislister?tidligere-versjoner#322595
12. Statens legemiddelverk. Price- and Reimbursement list. Updated January 6, 2022. Accessed March 19, 2022, https://legemiddelverket.no/english/public-funding-and-pricing/maximum-price#list-of-products-with-maximum-prices
13. Mikkelsen MR, Nielsen SB, Stage E, Mathiesen ER, Damm P. High maternal HbA1c is associated with overweight in neonates. *Dan Med Bull*. 2011;58(9):A4309.
14. Helsedirektoratet. *Endring i fødepopulasjon og konsekvenser for bemanning og finansieringssystem*. 2020. Accessed March 22, 2022. https://www.helsedirektoratet.no/rapporter/endring-i-fodepopulasjon-og-konsekvenser-for-bemanning-og-finansieringssystem/Rapport%20om%20f%C3%B8depopulasjonen.pdf/_/attachment/inline/3435df20-ea13-4d9f-99ed-f711d6ffbef0:51f3f1f4a94cd0893d94f09f3c7663d150ae61b0/Rapport%20om%20f%C3%B8depopulasjonen.pdf
15. Norsk gynekologisk forening. Veileder i fødselshelp. Accessed March 22, 2022. https://www.legeforeningen.no/foreningsledd/fagmed/norsk-gynekologisk-forening/veiledere/veileder-i-fodselshjelp/
16. Data from: Medical Birth Registry [public database]. 2021.
17. Shinar S, Melamed N, Abdulaziz KE, et al. Changes in rate of preterm birth and adverse pregnancy outcomes attributed to preeclampsia after introduction of a refined definition of preeclampsia: A population-based study. *Acta Obstet Gynecol Scand*. 2021;100(9):1627-1635. doi:10.1111/aogs.14199

18. Bernitz S, Aas E, Oian P. Economic evaluation of birth care in low-risk women. A comparison between a midwife-led birth unit and a standard obstetric unit within the same hospital in Norway. A randomised controlled trial. *Midwifery*. 2012;28(5):591-599. doi:10.1016/j.midw.2012.06.001
19. Senter for klinisk dokumentasjon og evaluering. *The Norwegian Neonatal Healthcare Atlas, 2009-2014*. 2016. Accessed April 12, 2022. https://helseatlas.no/sites/default/files/norwegian-neonatal-healthcare.pdf
20. Helsedirektoratet. Follow-up care during the post-natal period. Updated October 29, 2019. Accessed April 14, 2022. https://www.helsenorge.no/en/etter-fodsel/follow-up-care-during-the-post-natal-period/
21. Statistics Norway. 11418: Yrkesfordelt månedslønn, etter statistikkmål, yrke, statistikkvariabel og år. Accessed March 16, 2022. https://www.ssb.no/statbank/table/11418/
22. Statistics Norway. Konsumprisindeks, historisk serie fra 1924 (2015=100). Accessed March 19, 2022. https://www.ssb.no/priser-og-prisindekser/konsumpriser/statistikk/konsumprisindeksen
23. Jansen AJ, Essink-Bot ML, Duvekot JJ, van Rhenen DJ. Psychometric evaluation of health-related quality of life measures in women after different types of delivery. *J Psychosom Res*. 2007;63(3):275-81. doi:10.1016/j.jpsychores.2007.06.003
24. Rowen D, Brazier J, Roberts J. Mapping SF-36 onto the EQ-5D index: how reliable is the relationship? *Health and Quality of Life Outcomes*. 2009;7:27. doi:10.1186/1477-7525-7-27
25. Stern C, Trapp EM, Mautner E, Deutsch M, Lang U, Cervar-Zivkovic M. The impact of severe preeclampsia on maternal quality of life. *Qual Life Res*. 2014;23(3):1019-1026. doi:10.1007/s11136-013-0525-3
26. Lawrence WF, Fleishman JA. Predicting EuroQoL EQ-5D preference scores from the SF-12 Health Survey in a nationally representative sample. *Med Decis Making*. 2004;24(2):160-169. doi:10.1177/0272989X04264015
27. Walker KF, Dritsaki M, Bugg G, et al. Labour induction near term for women aged 35 or over: an economic evaluation. *BJOG*. 2017;124(6):929-934. doi:10.1111/1471-0528.14557
28. Ramage L, Yen C, Qiu S, et al. Does a missed obstetric anal sphincter injury at time of delivery affect short-term functional outcome? *Ann R Coll Surg Engl*. 2018;100(1):26-32. doi:10.1308/rcsann.2017.0140
29. Culligan PJ, Myers JA, Goldberg RP, Blackwell L, Gohmann SF, Abell TD. Elective cesarean section to prevent anal incontinence and brachial plexus injuries associated with macrosomia—a decision analysis. *Int Urogynecol J Pelvic Floor Dysfunct*. 2005;16(1):19-28. doi:10.1007/s00192-004-1203-3
30. Carroll AE, Downs SM. Improving decision analyses: parent preferences (utility values) for pediatric health outcomes. *J Pediatr*. 2009;155(1):21-25. doi:10.1016/j.jpeds.2009.01.040
31. Kwon J, Kim SW, Ungar WJ, Tsiplova K, Madan J, Petrou S. A Systematic Review and Meta-analysis of Childhood Health Utilities. *Med Decis Making*. 2018;38(3):277-305. doi:10.1177/0272989X17732990
32. Thomas S, Stephens L, Mills TA, et al. Measures of anxiety, depression and stress in the antenatal and perinatal period following a stillbirth or neonatal death: a multicentre cohort study. *BMC Pregnancy Childbirth*. 2021;21(1):818. doi:10.1186/s12884-021-04289-0
33. Wan X, Wang W, Liu J, Tong T. Estimating the sample mean and standard deviation from the sample size, median, range and/or interquartile range. *BMC Med Res Methodol*. 2014;14:135. doi:10.1186/1471-2288-14-135
34. Sorensen M, Arneberg F, Line TM, Berg TJ. Cost of diabetes in Norway 2011. *Diabetes Res Clin Pract*. 2016;122:124-132. doi:10.1016/j.diabres.2016.10.012
35. Sullivan PW, Slejko JF, Sculpher MJ, Ghushchyan V. Catalogue of EQ-5D scores for the United Kingdom. *Med Decis Making*. 2011;31(6):800-804. doi:10.1177/0272989X11401031
36. Statistics Norway. 05375: Life expectancy, by sex and age 1986 - 2021 Accessed May 19, 2022. https://www.ssb.no/en/statbank/table/05375/

37. Franco OH, Steyerberg EW, Hu FB, Mackenbach J, Nusselder W. Associations of Diabetes Mellitus With Total Life Expectancy and Life Expectancy With and Without Cardiovascular Disease. *Arch Intern Med*. 2007;167(11):1145-1151. doi:10.1001/archinte.167.11.1145
38. Brown J, Alwan NA, West J, et al. Lifestyle interventions for the treatment of women with gestational diabetes. *Cochrane Database Syst Rev*. 2017;2017(5)doi:10.1002/14651858.CD011970.pub2
39. Norwegian Institute of Public Health. *Guidelines for the submission of documentation for single technology assessments (STAs) of medical devices and diagnostic interventions*. 2021. Accessed January 19, 2022. https://www.fhi.no/globalassets/guidelines-for-the-submission-of-documents-for-stas-2021.pdf
40. Pillay J, Donovan L, Guitard S, et al. *Screening for Gestational Diabetes Mellitus: A Systematic Review to Update the 2014 U.S. Preventive Services Task Force Recommendation*. 2021. AHRQ Publication No. 21-05273-EF-1. https://www.ncbi.nlm.nih.gov/books/NBK573100/pdf/Bookshelf_NBK573100.pdf
41. Koivunen S, Viljakainen M, Mannisto T, et al. Pregnancy outcomes according to the definition of gestational diabetes. *PLoS One*. 2020;15(3):e0229496. doi:10.1371/journal.pone.0229496
42. Koning SH, van Zanden JJ, Hoogenberg K, et al. New diagnostic criteria for gestational diabetes mellitus and their impact on the number of diagnoses and pregnancy outcomes. *Diabetologia*. 2018;61(4):800-809. doi:10.1007/s00125-017-4506-x
43. O'Sullivan EP, Avalos G, O'Reilly M, et al. Atlantic Diabetes in Pregnancy (DIP): the prevalence and outcomes of gestational diabetes mellitus using new diagnostic criteria. *Diabetologia*. 2011;54(7):1670-1675. doi:10.1007/s00125-011-2150-4
44. Persson M, Fadl H, Hanson U, Pasupathy D. Disproportionate body composition and neonatal outcome in offspring of mothers with and without gestational diabetes mellitus. *Diabetes Care*. 2013;36(11):3543-3548. doi:10.2337/dc13-0899
45. Esakoff TF, Cheng YW, Sparks TN, Caughey AB. The association between birthweight 4000 g or greater and perinatal outcomes in patients with and without gestational diabetes mellitus. *Am J Obstet Gynecol*. 2009;200(6):672.e1-672.e4. doi:10.1016/j.ajog.2009.02.035
46. Husereau D, Drummond M, Augustovski F, et al. Consolidated Health Economic Evaluation Reporting Standards 2022 (CHEERS 2022) statement: updated reporting guidance for health economic evaluations. *BMC Med*. Jan 12 2022;20(1):23. doi:10.1186/s12916-021-02204-0
